# Decision-making and related outcomes of patients with complex care needs in primary care settings: a systematic literature review with a case-based qualitative synthesis

**DOI:** 10.1186/s12875-022-01879-5

**Published:** 2022-11-09

**Authors:** Mathieu Bujold, Pierre Pluye, France Légaré, the Participatory Review Team, Quan Nha Hong, Marie-Claude Beaulieu, Paula L. Bush, Yves Couturier, Reem El Sherif, Justin Gagnon, Anik Giguère, Genevieve Gore, Serge Goulet, Roland Grad, Vera Granikov, Catherine Hudon, Edeltraut Kröger, Irina Kudrina, Christine Loignon, Marie-Therese Lussier, Marie-Eve Poitras, Rebekah Pratt, Benoît Rihoux, Nicolas Senn, Isabelle Vedel, Michel Wensin

**Affiliations:** 1 Department of Management, Evaluation and Health Policy, School of Public Health, Université de Montréal, Montreal, Canada; 2 Department of Family Medicine, McGill University, Montreal, Canada; 3 Department of Family Medicine and Emergency Medicine, Université Laval, Quebec City, Canada

**Keywords:** Patients with complex care needs, Decisional needs, Primary care, Shared decision-making, Patient-practitioner communication, Interprofessional coordination, Vulnerable population, Multimorbidity, Polypharmacy

## Abstract

**Background:**

In primary care, patients increasingly face difficult decisions related to complex care needs (multimorbidity, polypharmacy, mental health issues, social vulnerability and structural barriers). There is a need for a pragmatic conceptual model to understand decisional needs among patients with complex care needs and outcomes related to decision. We aimed to identify types of decisional needs among patients with complex care needs, and decision-making configurations of conditions associated with decision outcomes.

**Methods:**

We conducted a systematic mixed studies review. Two specialized librarians searched five bibliographic databases (Medline, Embase, PsycINFO, CINAHL and SSCI). The search strategy was conducted from inception to December 2017. A team of twenty crowd-reviewers selected empirical studies on: (1) patients with complex care needs; (2) decisional needs; (3) primary care. Two reviewers appraised the quality of included studies using the Mixed Methods Appraisal Tool. We conducted a 2-phase case-based qualitative synthesis framed by the Ottawa Decision Support Framework and Gregor’s explicative-predictive theory type. A decisional need case involved: (a) a decision (what), (b) concerning a patient with complex care needs with bio-psycho-social characteristics (who), (c) made independently or in partnership (how), (d) in a specific place and time (where/when), (e) with communication and coordination barriers or facilitators (why), and that (f) influenced actions taken, health or well-being, or decision quality (outcomes).

**Results:**

We included 47 studies. Data sufficiency qualitative criterion was reached. We identified 69 cases (2997 participants across 13 countries) grouped into five types of decisional needs: ‘prioritization’ (*n =* 26), ‘use of services’ (*n =* 22), ‘prescription’ (*n =* 12), ‘behavior change’ (*n =* 4) and ‘institutionalization’ (*n =* 5). Many decisions were made between clinical encounters in situations of social vulnerability. Patterns of conditions associated with decision outcomes revealed four decision-making configurations: ‘well-managed’ (*n =* 13), ‘asymmetric encounters’ (*n =* 21), ‘self-management by default’ (*n =* 8), and ‘chaotic’ (*n =* 27). Shared decision-making was associated with positive outcomes. Negative outcomes were associated with independent decision-making.

**Conclusion:**

Our results could extend decision-making models in primary care settings and inform subsequent user-centered design of decision support tools for heterogenous patients with complex care needs.

**Supplementary Information:**

The online version contains supplementary material available at 10.1186/s12875-022-01879-5.

## Background

Primary care plays a crucial role regarding patients with complex care needs (PCCNs) [1, 2]. Typically, PCCNs combine multimorbidity, polypharmacy, mental health issues, and social vulnerability, and face structural barriers that hamper optimal use of health and social care services [3–5]. PCCNs experience unmet care needs, fragmented care, inadequate quality of care, poor health outcomes, and inappropriate services overuse or underuse [6, 7]. This population is often faced with difficult decisions, e.g., to visit emergency department or to use home care services [8, 9]. These decisions are often complicated by a lack of consensus between the stakeholders (patient, caregiver, and practitioners). In line with Luhmann [10, 11], complexity refers to the repeated pressure on PCCNs, their caregivers and practitioners to select one option among many, despite uncertainties and lack of consensus undermining the decision-making process.

In this article, decisional needs are situational, i.e., they refer to the context of the decision and the experience of the stakeholders. Many PCCNs experience decisional conflict which can be marked by a state of individual uncertainty regarding decisions that challenge personal values [12]. Uncertainty in decision-making can lead to negative decision outcomes, e.g., delay decision, overwhelmed patient, inappropriate use of health services [13]. Unmet decisional needs, such as inadequate knowledge or unclear values, can exacerbate this uncertainty [12]. In turn, unmet needs might lead to negative emotions such as decision regret, affect healthcare use (e.g., provoke inappropriate overuse or underuse of services), and produce harmful health outcomes (e.g., harms of non-beneficial options). Decision support tools and interventions that address decisional needs may improve decision quality, i.e., are congruent with a patient’s [or caregiver’s] personal values and informed by empirical evidence [14]. Improving decision quality may lead to positive outcomes, i.e., reduce delays in decision-making, the inappropriate use of services, and negative emotions such as blame or regret [13].

Decisional needs assessments aim to describe the context of decision-making and the specific support required. Assessing the decisional needs of patients focusses on identifying specific decision points with multiple options [15, 16]. Decision support interventions aim to address decisional needs by (a) resolving decisional conflict, (b) improving knowledge and information exchange, (c) clarifying patient values, preferences, and expectations, and (d) identifying the resources they need including social support. PCCNs face cascade of difficult decisions involving multiple stakeholders (caregivers and practitioners). Meeting decisional needs and reducing the harm caused by poor decision quality is even more urgent for them. However, there are significant knowledge gaps regarding the decisional needs of PCCNs and their caregivers [17]. In addition, no study has previously outlined possible downstream outcomes of decisions made by or for PCCNs. These gaps hinder the design of adapted decision support for this population. Therefore, we aimed to identify types of decisional needs among PCCNs, and patterns of decision-making conditions associated with decision outcomes (hereafter, decision-making configurations).

## Methods

### Study design

The protocol was previously published [18]. We conducted a systematic mixed studies review (qualitative, quantitative, and mixed methods studies) with an international multidisciplinary team including primary care researchers and knowledge users (patient-partner, practitioners, service managers and policy makers) [19, 20]. We systematically searched, identified, selected, appraised, and synthesized qualitative and quantitative evidence. Since there is no reporting guideline for systematic mixed studies reviews, this review combined the reporting recommendations from the PRISMA statement for systematic literature reviews [21] and the ENTREQ statement for enhancing transparency in reporting a qualitative review [22] (PRISMA checklist and addressed elements of the ENTREQ statement are available in additional file 1).

### Eligibility criteria

We based eligibility criteria on a pilot project, a case series [8] and a scoping review [9], that sought to identify characteristics of PCCNs and possible support interventions. We included empirical studies (using qualitative, quantitative or mixed methods) written in French, English or Spanish when they (1) dealt directly or indirectly with PCCNs, or a population with at least two of the following characteristics: multimorbidity; mental health issues; polypharmacy; social vulnerability (socio-economic deprivation, highly disabled people, marginalized populations); or healthcare services overuse or underuse, (2) concerned a decision-making process and included a decision outcome description (i.e. actions taken or well-being impacts); (3) were conducted in a primary care setting or dealt with primary care settings directly or indirectly, e.g., transition from hospital. Because of the specificity of their decisional needs (e.g., the end-of-life situation), we excluded two populations: people in palliative care and children with complex care needs.

### Search strategy

Two specialized librarians in systematic mixed studies reviews developed and tested the search strategy (available in additional file 2) to include the four main concepts: PCCNs, decisional needs, primary healthcare, and empirical research. They explored five bibliographic databases: Medline (Ovid), Embase (Ovid), PsycINFO (Ovid), CINAHL (EBSCOhost), and the Social Sciences Citation Index (Web of Science). In each database, the search strategy was conducted from inception to December 2017. In addition, we asked the Participatory Review Team members to point us to any other relevant studies.

### Selection of eligible studies

We trained twenty crowd-reviewers to select relevant studies. This method of selection by crowdsourcing is detailed elsewhere [23, 24].

We imported bibliographic records (including titles and abstracts) into EndNote and removed duplicates using the Bramer’s method [25]. Then, we imported all studies into specialized online software for selection (DistillerSR). Crowd-reviewers were researchers, graduate students, and practitioners from different disciplines (family medicine, nursing, occupational therapy, epidemiology, and social sciences). Seven crowd-reviewers were Participatory Review Team members. The lead author (A1) trained the crowd-reviewers to include and exclude studies using DistillerSR. All imported studies were screened by at least two independent reviewers. In addition, the trainer met crowd-reviewers individually during the selection process (monitoring) and clarified inclusion and exclusion criteria when needed. Based on this selection process, with continuous quality control, the interdisciplinary diversity of crowd-reviewers’ viewpoints helped us establish an initial raw classification of PCCNs- and decisional needs-related concepts. For each record, two independent reviewers performed the following two-step selection:

Step 1: For each title/abstract, two crowd-reviewers independently answered four questions: (a) Is this record about a study involving PCCNs?; (b) Is this record useful for decisional needs assessment?; (c) Does this record involve (deal with) a primary health care setting?; and (d) Is this an empirical study? The response options were: yes, no, cannot tell. A1 made the final decision concerning records with contradictory responses.

Step 2: For each full-text paper, two crowd-reviewers independently applied the same eligibility criteria and response options. A1 trained crowd-reviewers using a comprehensive codebook with definitions and illustrative examples. The codebook helped reviewers to quickly justify the inclusion of a study.

### Critical appraisal of included studies

We used the Mixed Methods Appraisal Tool (MMAT) to appraise and describe the methodological quality of the included qualitative, quantitative and mixed methods studies [26]. Two reviewers (a lead author and a member of the Participatory Review Team) independently appraised all included studies and met to resolve disagreements (no third-party arbitration was needed).

### Data extraction and qualitative synthesis

A1 extracted the characteristics of included studies (year, research design and number of PCCNs) and excerpts on decisional needs. The second author (A2) verified the extraction. With respect to the few studies that compared PCCNs with other patients, we retained only the PCCNs-related data. Then we performed a hybrid deductive/inductive thematic synthesis of the qualitative evidence [27] in two phases [20] to build a typology of decisional needs and decision-making configurations.

Qualitative methodologies are appropriate to get an in-depth and holistic understanding of complex phenomena [28], like PCCNs’ decisional needs. Data sufficiency is the criterion used to appraise reviews with qualitative synthesis: “(1) data should be sufficient to permit comparisons among selected dimensions and constructs; (2) the reports should reflect the work of several distinct and independent investigators; and (3) the data should be sufficient to answer the research question” (29: p. 37 cited in [29]).

In addition, literature reviews with qualitative syntheses give an essential place to theories [30], and can combine qualitative and quantitative evidence [20].

#### Phase 1 –typology of decisional needs

With respect to the hybrid synthesis, the deductive component of the synthesis was derived from the Ottawa Decision Support Framework (ODSF) [31]. The ODSF workbook proposes a step-by-step guide for decisional needs assessment that can be performed using different data collection methods, including a review of the literature [16].

According to the ODSF workbook, our initial codebook included the following thematic categories: (a) type of decision; (b) PCCNs’ bio-psycho-social characteristics; (c) stakeholders’ roles in the decision-making; (d) decision-making context; (e) intrinsic or extrinsic conditions influencing decision-making; (f) decision outcomes (e.g., actions taken, health and well-being impacts, and decision quality, i.e., informed and/or value-based). In addition, the decision support tools and strategies were codified.

We conducted data extraction, interpretation, and coding iteratively, going back and forth from articles and data to themes. A1 coded articles using NVivo 11 [32], and made an inventory of decisional needs with written interpretations. A1 and A2 ensured consistency and rigor by holding weekly meetings and combining their interpretations of the decisional needs identified [33].

Then, in the inductive component, A1 and A2 identified themes and subthemes suggested by the data. They performed the synthesis and produced an initial codebook including a structured list of these themes and subthemes with definitions and illustrative examples. In 2017, in the project design step, they had conducted four meetings with a core group of 12 co-researchers including a patient-partner and three members of an international advisory committee [18]. This led them to revise the codebook (available on request), clarifying the definitions and adding subthemes. In 2018, they conducted seven group meetings with the Participatory Review Team members who discussed, approved, or improved Phase 1 results and Phase 2 methodological elements.

#### Phase 2 – decision-making configurations

A1 used NVivo’s advanced queries functions (qualitative matrix) to cross-analyze the themes and establish relationships among them. Following Gregor’s explicative-predictive theory type, we synthesized cases to explain decisional needs and predict testable propositions of pattern of decision-making conditions associated with decision outcomes [34]. In line with Gregor [34], an explanation and prediction theory aims to answer the following question: what is, how, why, when, where, and what will be (e.g. outcome). We defined a case as a decisional need involving: (a) a decision, e.g. use of service (what), (b) concerning a PCCN with bio-psycho-social characteristics, e.g. social vulnerability (who), (c) made independently or in partnership, e.g. with the practitioner (how), (d) in a specific place and time, e.g. during a clinical encounter (where/when), (e) with communication and coordination barriers or facilitators, e.g. communication quality with practitioners or interprofessional team (why), (f) an influence on actions taken, health and well-being, decision quality (decision outcomes)*.*

For each case, A1 wrote a memo, i.e., a summary of the case-related data and themes, explaining decision-making configurations, i.e., the conditions (what, who, how, where, when, why) and related outcomes (6 W-O). In one article, there may be several cases (memos) illustrating distinct conditions leading to different decision outcomes. When outcomes reported in included studies were clearly negative (or positive), the corresponding outcomes were attributed in the configurations. When the reported outcomes were ambiguous, the outcomes of the decision were assessed as equivocal in the configurations.

**Fig. 1:**
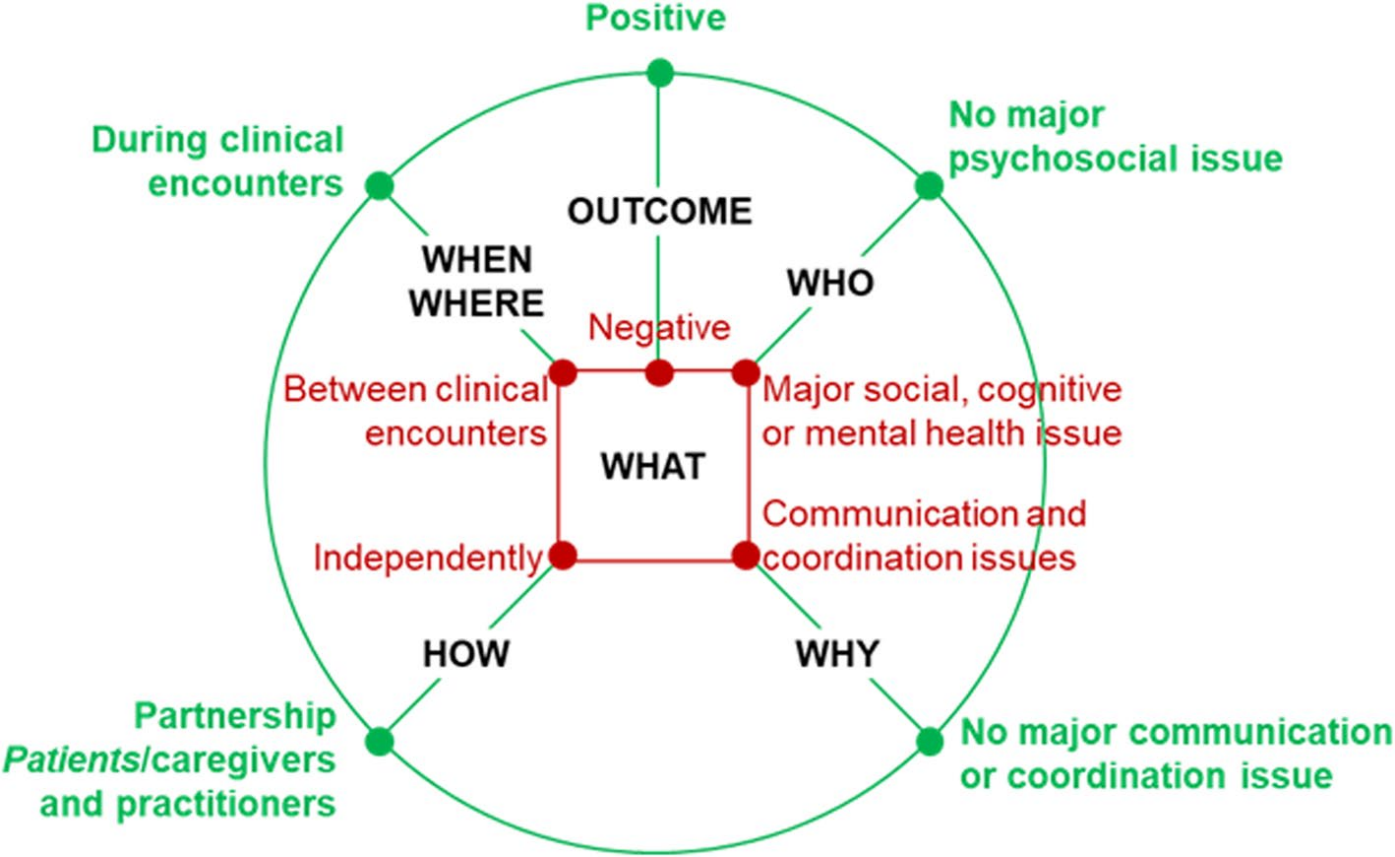
Decisional needs of patients with complex care needs: Template diagram for data visualization

These memos were reviewed and discussed by A1 and A2 in weekly meetings and illustrated with a visual diagram in the form of a spider web representing the 6 W-O relationships of each case (Fig. 1). Spider web diagrams are a visualization tool that helps compare different dimensions of a complex concept [29]. To illustrate the types of decisional needs identified, we selected 31 typical memos (maximum 6 W-O variation sampling) and discussed them with five knowledge users of the Participatory Review Team: a social worker, a nurse, a pharmacist and two family doctors. The knowledge users validated the cases based on their expertise and practical experience. Second, we mapped each case on to our spider-web diagram, cross-associating similar outcomes, and conditions to create a series of different patterns. We then grouped these patterns into decision-making configurations.

## Results

### Description of included studies

**Fig. 2:**
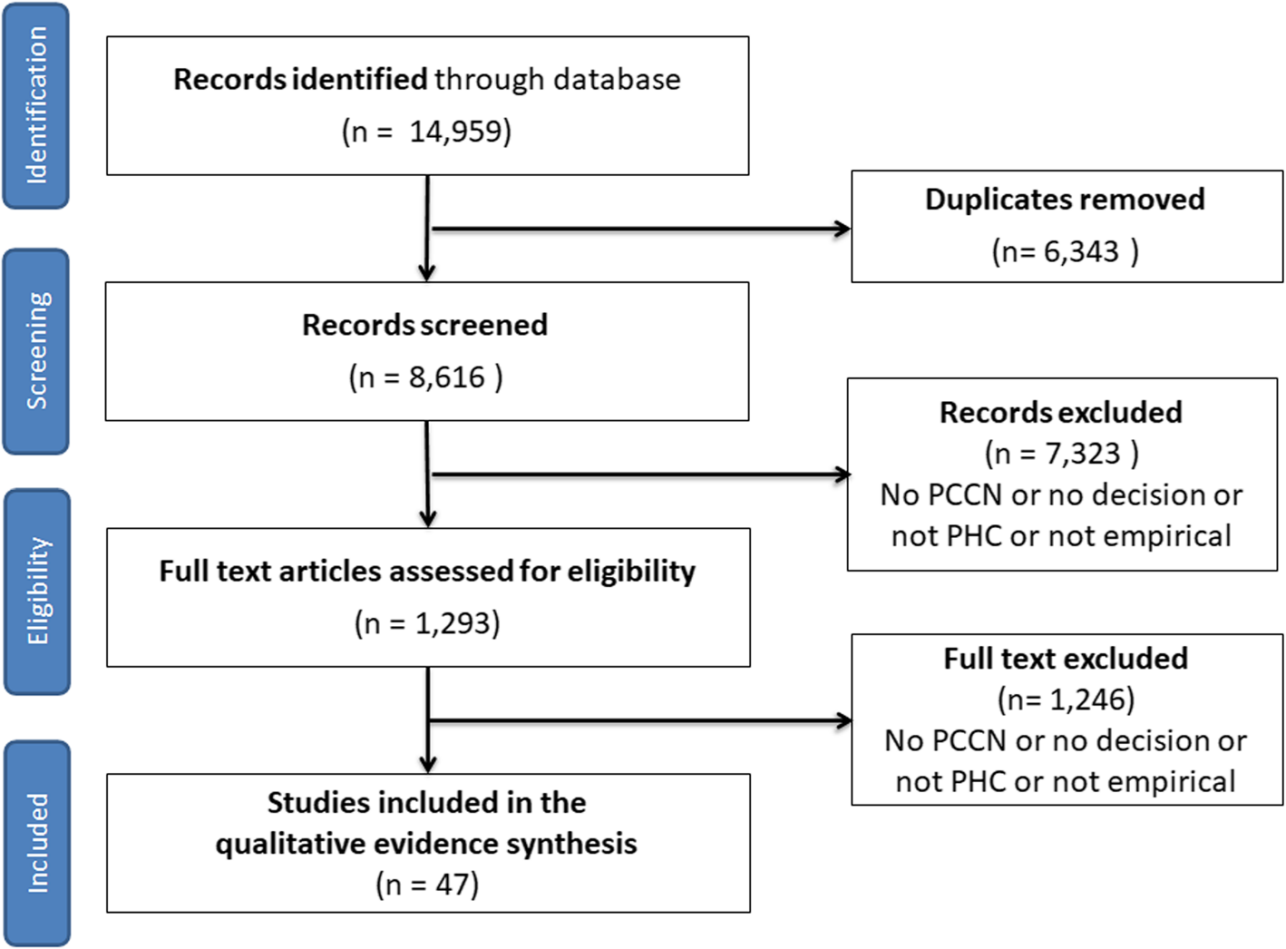
Flow Chart

As shown in Fig. 2, we screened 8616 records and 1293 full-text papers. Of those, 47 studies were included in the synthesis: 41 qualitative, two quantitative and four mixed methods studies (Table 1). Together, these studies represent a large sample of 2997 participants (2107 patients, 698 practitioners and 192 caregivers) from 13 countries. Among the studies, we identified 69 decisional needs cases (see additional file 3 tables for more details about cases).

**Table 1:** Table of characteristics of included studies (*n =* 47)

Sources	# of participants (total)	# of patients	# of caregivers	# of practitioners	Country
[35]	8	0	0	8	Australia
[36]	26	0	0	26	Australia
[37]	20	0	20	0	Australia
[38]	26	26	0	0	Australia
[39]	807	807	0	0	Belgium
[40]	33	25	8	0	Canada
[41]	59	27	27	5	Canada
[42]	35	35	0	0	Canada
[43]	23	0	17	6	Canada
[44]	65	16	37	12	Canada
[45]	81	29	24	28	Canada
[46]	62	62	0	0	Canada
[47]	83	27	28	28	Canada
[48]	36	21	0	15	Germany
[49]	86	43	0	43	Germany
[50]	21	21	0	0	Ireland
[51]	41	28	13	0	Lebanon
[52]	25	0	0	25	Netherlands
[53]	73	60	0	13	Netherlands
[54]	22	11	11	0	Netherlands
[55]	142	0	0	142	Norway
[56]	16	0	0	16	New Zealand
[57]	180	0	0	180	Sweden
[58]	16	4	0	12	Sweden
[59]	10	5	0	5	Switzerland
[60]	41	41	0	0	United Kingdom
[61]	32	19	0	13	United Kingdom
[62]	14	14	0	0	United Kingdom
[63]	40	20	0	20	United Kingdom
[64]	18	18	0	0	United Kingdom
[65]	7	0	7	0	United Kingdom
[66]	40	20	0	20	United Kingdom
[67]	55	35	0	20	United Kingdom
[68]	37	8	0	29	United Kingdom
[69]	50	50	0	0	United Kingdom
[70]	28	28	0	0	United Kingdom
[71]	30	30	0	0	United Kingdom
[72]	302	302	0	0	United States
[73]	57	40	0	17	United States
[74]	53	53	0	0	United States
[75]	50	50	0	0	United States
[76]	30	30	0	0	United States
[77]	21	21	0	0	United States
[78]	20	20	0	0	United States
[79]	25	25	0	0	United States
[80]	36	36	0	0	United States
[81]	15	0	0	15	United States

Overall, the quality of the 47 included studies was deemed good, according to the 2018 version of the MMAT (see additional file 4). Among the 41 qualitative studies, 14 addressed all five MMAT qualitative criteria, 23 did not specify the qualitative design (one criterion unmet), and four did not meet one or more criteria regarding data collection, analysis, interpretation, or coherence. One non-randomized study did not meet the measurement criterion and one descriptive quantitative study (descriptive statistics on baseline data of a trial) did not meet the criterion on sampling and sample representativeness. Of the four mixed methods studies, two met the five MMAT mixed methods criteria, one presented an adequate rationale for using a mixed methods design to address the research question, and one did not adequately integrate the various components of the study to answer the research question or address divergences and inconsistencies.

### Decisional needs typology

This section presents the emergent (suggested by data) typology of decisional needs, grouped in five types of decision: ‘prioritization’ (*n =* 26 cases), ‘use of services’ (*n =* 22 cases), ‘prescription’ (*n =* 12 cases), ‘behaviour change’ (*n =* 4 cases), and ‘institutionalization’ (*n =* 5 cases) (Table 2). These thematic categories correspond to the following main questions, respectively: Which issues should the patient prioritise? Should the patient consult or not? Should the practitioner prescribe or deprescribe, or should the patient adhere or not to the prescription? Does the patient stop or maintain unhealthy lifestyle behaviour (e.g., smoking)? Caregiver’s question: Should my relative stay home or should they move to an institution (e.g., nursing home)? In addition to the type of decision, the next sections also provide a description of related decision-making conditions, including the use of tools and strategies.

**Table 2:** Five types of decisional needs

	Types of decisional needs				
Conditions linked to decisional needs	Prioritization (26 cases 1210 participants)	Use of services (22 cases; 769 participants)	Prescription (12 cases; 460 participants)	Behaviour change (4 cases; 267 participants)	Institutionalization (5 cases; 982 participants)
**Who** PCCN bio-psycho-social characteristics	multimorbidity (*n =* 23) polypharmacy (*n =* 9) social vulnerability (*n =* 11) mental health issues (*n =* 12) frequent user* (*n =* 1); under user (*n =* 2)*	multimorbidity (*n =* 17) polypharmacy (*n =* 9) social vulnerability (*n =* 16) mental health issues (*n =* 6) frequent user (*n =* 7); under user (*n =* 4)	multimorbidity (*n =* 12) polypharmacy (*n =* 11) social vulnerability (*n =* 9) mental health issues (*n =* 8) frequent user (*n =* 1) under user (*n =* 1)	multimorbidity (*n =* 1) polypharmacy (*n =* 1) social vulnerability (*n =* 4) mental health issues (*n =* 3) frequent users (*n =* 1); under users (*n =* 1)	multimorbidity (*n =* 2) polypharmacy (*n =* 1) social vulnerability (*n =* 5) mental health issues (*n =* 5)
**How** The decision is made in partnership or independently (decision driven by)	partnership (*n =* 9) independently (*n =* 17) (8 by practitioner; 9 by patient)	partnership (*n =* 4) independently (*n =* 18) (3 by practitioner; 13 by patient; 2 by caregiver)	all independently = (*n =* 12) (8 by practitioner; 4 by patient)	all independently = (*n =* 4) (3 by patient; 1 by caregiver)	all independently (*n =* 5) (4 by caregiver; 1 by patient)
**Where / When** The decision is made during or between clinical encounter	during (*n =* 19) between (*n =* 7)	during (*n =* 7) between (*n =* 15)	during (*n =* 8) between (*n =* 4)	all between (*n =* 4)	all between (*n =* 5)

*These participants report an inappropriate use of services (overuse or underuse)

#### Prioritization: needs to deal with competing priorities of PCCNs

##### Highlights

- Due to the combination of several issues (e.g., multimorbidity, polypharmacy, social vulnerability), patients are confronted with three interrelated levels of prioritization: Which discussion topic should I present in a time-limited clinical encounter? What health issues should I prioritize in my care? How do I negotiate my other life priorities compared to my care priorities?
- Most prioritizations are done independently by practitioners or patients.
- Patients and practitioners have different perceptions of priorities.
- Prioritization tools and strategies are occasionally used in partnership by patients and practitioners.

The most common decisional needs concerned the difficulty prioritizing mental, physical, social, or other issues (26 of 69 cases). We identified 26 ‘prioritization’ cases derived from 20 studies, which represented 1210 participants (740 patients, 55 caregivers and 415 practitioners). Cases concerned patients with overlapping bio-psycho-social characteristics: multimorbidity (*n =* 23/26), polypharmacy (*n =* 9/26), mental health issues (*n =* 12/26) and social vulnerability (*n =* 11/26). Prioritization occurred during (*n =* 19) or between (*n =* 7) clinical encounters.

In the context of multimorbidity, the patient often had a lot of information to share during clinical meetings. They had to decide which information to present during a time-limited clinical encounter. Then, the practitioner would choose from among the patient’s multiple concerns which topics to focus on. After an information exchange, the patient, and the practitioner, independently or in partnership, determined the priorities and goals to address.

In 10 cases, practitioners or patients prioritized independently during clinical encounters (see additional file 3.1). Of those, eight cases reported a lack of patient-practitioner communication. Typically, the practitioner led the discussion during the clinical encounter and selected the topics to be addressed (paternalistic prioritization). The patient realized that the practitioner was omitting certain issues, but decided to remain silent. Some practitioners complained about the overloaded agenda of PCCNs. For their part, patients sometimes selected the topics they wanted to discuss and deliberately avoided others that were clinically important, e.g., masking depression symptoms to avoid being given antidepressants (prioritization by omission). Other patients thought their point of view was trivial and feared that the practitioners would laugh at them.

In nine cases, practitioners and patients prioritized in partnership during the clinical encounter (see additional file 3.2). The prioritization was explicit, with discussion and decision-making on a few selected issues. In all nine cases, stakeholders used a prioritization tool or strategy. First, some patients used tools in preparation for clinical encounter, and used it with the practitioner during the encounter (e.g., a collaborative goal-setting aid). Second, they used another strategy if there was no tool available, e.g., choosing a few priorities beforehand to address in the clinical encounter, and scheduling several encounters to address all their priorities. Another strategy consisted of finding a balance between the practitioner’s disease-related priorities and the patient’s illness-related priorities during the clinical encounter. Patients tended to prioritize issues related to functional health, quality of life and autonomy, e.g., appropriate support for maintaining activities of daily living. In contrast, practitioners tended to prioritize issues related to the disease itself, e.g., the most severe health issues.

In seven cases, patients prioritized independently between clinical encounters (see additional file 3.3). Some socially vulnerable PCCNs felt they needed to prioritize activities of daily living over health care issues, e.g., marginalized people (homeless, drug users, migrants, sex workers). Some patients weighed the current and daily impact of some of their long-term conditions against those which they perceived could produce more serious and negative outcomes in the future. Some patients in situations of socio-economic deprivation decide to not adhere to care goals by inability to pay or a choice to prioritize daily living activities over care.

#### Use of services: needs to decide whether the use of services is appropriate

##### Highlights

- Patients with multimorbidity and in a situation of social vulnerability are confronted with different decision related to use of services: Should I consult or not? Should I use home care or social services, or not? Should I engage or not in a program? Should I adhere to an intervention or not? When should I go to the hospital or the emergency room?
- Patients and practitioners have different perceptions of what is a situation of frequent use of services.
- Some patients in situation of social vulnerability inappropriately underuse health services due to limited access or service refusal.
- Most use of service-related decisions were made independently by patients and caregivers between clinical encounters.
- Tools are sometimes used in partnership with patients and practitioners to guide decisions on the use of services.

Another common type of decisional needs concerned use of services (22 of 69 cases). The 22 cases, derived from 19 studies, represented 765 participants (463 patients, 52 caregivers and 250 practitioners). Patients with this type of decisional need were in situations of multimorbidity (*n =* 17/22), social vulnerability (*n =* 16/22) and faced problems with patient-practitioner communication (*n =* 15/22) and interprofessional coordination (*n =* 8/22).

In seven cases, practitioners perceived these patients as frequent users of services, although their patients did not share this perception. Patients reported that they used services when their self-management burden threatened to overwhelm them. Four cases concerned patients in situations of social vulnerability who underused health services due to limited access or service refusal, e.g., marginalized people.

In 15 ‘use of services’ cases, decisions were made independently, mostly by patients, between clinical encounters (see additional file 3.4). The patients decided independently in 14 cases and the practitioners in one case (exclusion of a patient from a program). Patients were mostly in situations of social vulnerability (10 of 14 cases). Twelve cases explained the difficulty of the decision-making due to the lack of patient-practitioner communication (*n =* 10) and a lack of inter-professional coordination (*n =* 7). These 15 ‘use of services’ cases concerned consultations with practitioners (*n =* 6), social and home care services (*n =* 4), emergency room or hospitalization (*n =* 2), engagement in a program (*n =* 2) and interventions (*n =* 1). Regarding consultations, some patients preferred self-management and refused services, while others were lost in the complexity of services or felt abandoned. With respect to social and home care services, patients’ and caregivers’ decisional needs fluctuated over time depending on the evolution of patients’ multimorbidity and financial resources, or on their social support. Typically, elderly patients felt overwhelmed, overestimated their autonomy, and refused or delayed social and home care services. Regarding hospital services, patients reported the emergency room and hospitalization as last resort options when the burden of self-management exceeded their capacities. None of these 15 cases reported use of a decision-making tool. Two cases reported strategies (see additional file 3.4) that could be developed further such as online communication technologies (e.g., secure messaging and video-conferencing) to aid self-management and receive specialized support (e.g., a social worker) for navigating health and social services.

Three ‘use of services’ cases presented decisions made independently during clinical encounters (see additional file 3.5). Decisions were made independently by the physicians in two cases, and by patients or caregivers in one. All cases displayed a lack of patient/caregiver-practitioner communication, often associated with negative perceptions of the other stakeholders, perhaps due to an unpleasant previous interaction. Some patients and caregivers, for example, withheld health information, refused to comply with regulations, or declined assistance with activities of daily living for instance. Some practitioners felt uneasy with patients whom they saw as ‘difficult’ because they were non-compliant, frequent users of services, in situations of social vulnerability, time-consuming, or seriously ill and in need of high-intensity care. Some practitioners saw self-management as positive as it reduces use of services, while many patients considered seeing their doctor and nurse as a last resort, and their motivation to self-manage did not reflect a desire to reduce their use of healthcare services.

Four cases reported use of services decisions made in partnership with the practitioner during the clinical encounter (see additional file 3.6). In all four cases, stakeholders used a tool, a strategy, or a program to facilitate decision-making. One case included a tool for helping caregivers to select appropriate home care services: patients and caregivers had to decide (accept/refuse) among multiple service options. Two cases presented patient-centred strategies. One case presented a management program that established individualized care plans for patients identified by their physicians as frequent users.

#### Prescription: needs to manage drug-related decisions in a poor patient-practitioner communication context

##### Highlights

- Practitioners and their patients in a situation of multimorbidity, polypharmacy and social vulnerability are confronted decision related to prescription.
- Prescription decision was taken independently, mostly by practitioners (Should I prescribe or not? How to deprescribe?), but sometimes by patients (Should I adhere or not to prescription?).
- No case reported a decision-making tool.

Twelve cases out of 69 reported decisional needs related to medication prescription (hereafter, prescription), which represented 460 participants including 176 patients, 35 caregivers and 249 practitioners. Patients faced multimorbidity, had polypharmacy and were in situations of social vulnerability (or all three) in 12, 11 and 10 cases, respectively. In all ‘prescription’ cases, the patients or practitioners decided independently. Eight cases presented a lack of inter-professional coordination and six cases a lack of patient-practitioner communication.

In eight cases, ‘prescription’ decisional needs concerned practitioners’ decisions made independently during clinical encounters (see additional file 3.7). The eight cases concerned prescribing vs no-prescription (*n =* 5) or deprescribing (*n =* 3). None of these cases reported a decision-making tool, while two mentioned strategies to improve decision-making processes (see additional file 3.7). In one strategy, practitioners presented the positive and negative effects of the medication to the patient. In another strategy, patients enhanced the quality and amount of information provided by practitioners by bringing someone to the clinical encounter, preparing the encounter with a list of questions written in advance, reporting things mentioned previously by other practitioners, and searching information by themselves in diverse sources, e.g., the Internet.

In four cases, ‘prescription’ decisional needs concerned patients’ decisions made independently, between clinical encounters (see additional file 3.8). Patients chose on their own not to adhere to prescriptions (medication not bought, or not taken). Some patients in situations of polypharmacy and lack of interprofessional coordination (e.g., too many prescribers) decided to stop or reduce medication by fear of iatrogenic drug-drug interaction. In all cases, patients were in situations of social vulnerability and faced mental health issues.

#### Behavior change: needs to deal with unhealthy lifestyles in the context of social vulnerability and poor patient-practitioner communication

##### Highlights

- Patients in situation of social vulnerability and their caregivers are confronted to behavior change decision
- Behavior change decision was made independently by patients between clinical encounters.
- No case reported a behavior change-oriented decision-making tool.

Four cases reported decisional needs pertaining to behavior change (see additional file 3.9), and corresponded to 267 participants including 80 patients, 40 caregivers and 147 practitioners. Two cases reported alcohol use, sedentary lifestyles and smoking by patients with chronic obstructive pulmonary diseases. In a third case, patients with severe mental disorders did not alter their unhealthy lifestyles. In the fourth case, elderly patients with dementia continued to drive. All cases involved patients in situations of social vulnerability, combined with mental health issues in three cases. All cases reported a lack of patient-practitioner communication and two cases a lack of inter-professional coordination. Patients or caregivers decided independently between clinical encounters. Patients and caregivers did not adhere to prescribed behavioral strategies.

#### Institutionalization: needs to handle conflictual decisions concerning the transfer of a cognitively impaired person to long term care

##### Highlights

- With respect to non-autonomous patients, some caregivers are confronted with family conflicts and overwhelmed by the institutionalization-related decision-making process.
- A critical incident often triggered ‘institutionalization’ decisional needs.
- No case reported a decision support tool.

Five cases reported decisional needs concerning transfer to long term care (hereafter institutionalization), which represented 982 participants including 850 patients, 92 caregivers and 40 practitioners (see additional file 3.10). These cases showed that decisional needs about this issue are emotional and sometimes heart breaking. Typically, they involved caregivers making decisions for elderly patients in situations of social vulnerability with cognitive/mental disorders such as dementia who are unable to participate in decision-making. Also specific to this type of decisional need was that all cases presented family conflicts, aggravated in two cases by migration-related and generational cultural conflict. Decision-making confronted cultural clashes such as (a) communitarian values (favoring home care managed by the family) versus individualistic values (favoring transfer to long term care), and/or (b) traditional values regarding gender-based roles in decision-making roles versus feminist values. Multiple reasons triggered ‘institutionalization’ decisional needs (unmanageable or unsafe patient behavior, uncontrolled chronic conditions, patients’ dependency on full-time care services, and caregiver burden). Two cases presented decision-making strategies (see additional file 3.10). In the first case, relatives and practitioners were convinced that institutionalization was needed, waited for an incident to make it happen, or disguised the institutionalization as short-term hospitalization. In the second case, patients, caregivers, and practitioners had to negotiate between conflicting cultural values (North American versus Asian) and created alliances at turning points in the patients’ lives (alliances between a wide variety of family members and health care and social care practitioners).

### Decision-making configurations: patterns of conditions associated to decision outcomes

**Fig. 3:**
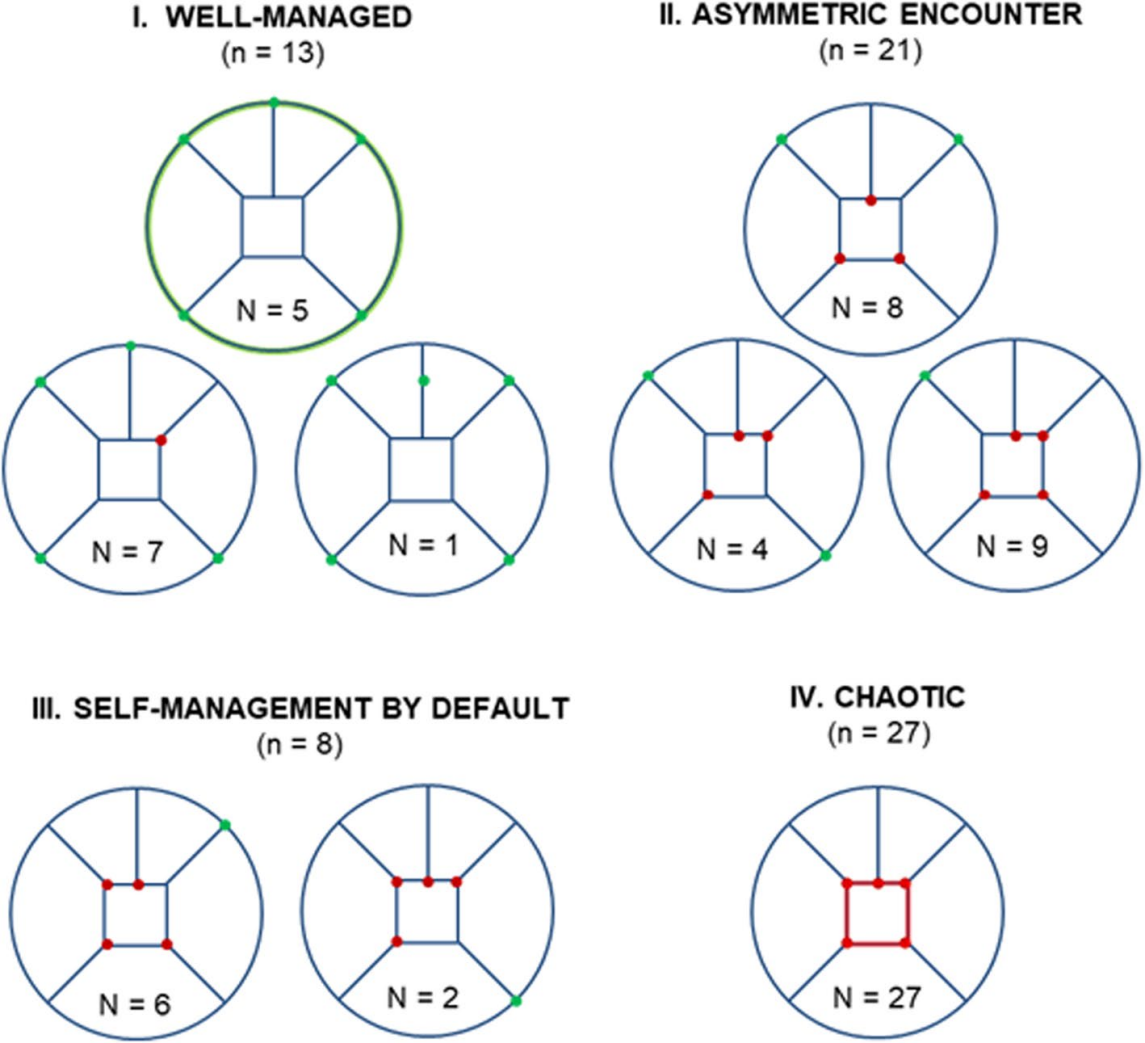
Four decision-making configurations in primary care

The visualization and interpretation of the 69 cases, documented by 6 W-O memos using spider web diagrams derived from the Fig. 1, revealed nine patterns of cases with similar outcomes and conditions. As shown in Fig. 3, we grouped patterns into four decision***-***making configurations: ‘well-managed’ (*n =* 13), ‘asymmetric encounters’ (*n* = 21), ‘self-management by default’ (*n* = 8), and ‘chaotic’ (*n* = 27).

#### Well-managed

The ‘well-managed’ configuration represented the best-case scenario. In this decision-making configuration, 13 cases sharing conditions and outcomes (6 W-O) grouped into three patterns (Fig. 3). Cases concerned decisional needs regarding ‘prioritization’ (*n =* 9) and ‘use of services’ (*n =* 4). Of the 13 cases, one had an equivocal outcome, and 12 reported positive outcomes. Positive outcomes were linked to actions taken (e.g., patient engagement in care plan; services use or access), to emotional impact (e.g., patient and caregiver experiencing positive emotion) and decision quality (informed and value-based). In this configuration, cases reported satisfactory patient-practitioner communication and interprofessional coordination between primary care services and other mental health and social care services, and decisions made in partnership by PCCNs, their caregivers and practitioners during clinical encounters. The Fig. 3 shows, for this configuration, two patterns of conditions associated with positive outcomes. Of 12 cases, five corresponded to the ‘full green circle’ pattern and concerned patients with no major psychosocial issues; and seven cases corresponded to the ‘almost full circle’ pattern concerning patients in situations of social vulnerability (e.g., socio-economic deprivation) or faced mental health issues (e.g., depression). Although some physicians reported that their patients’ psychosocial issues were challenging, their conditions were nevertheless well-managed. Decision outcomes were equivocal in one case because sharing a decision about health priorities seemed unusual for both the doctors and elderly patients. All cases reported a decision-oriented tool (*n =* 5), strategy (*n =* 7), or program (*n =* 1).

#### Asymmetric encounters

In the ‘asymmetric encounters’ configuration, 21 cases sharing similar conditions grouped into three patterns (Fig. 3). This decision-making configuration exposed informational and power asymmetries during clinical encounters. Practitioners decided independently in 17 cases (e.g., prescribed or deprescribed a drug), and patients made independent decisions in the four other cases (e.g., decisions about what information to prioritize for presenting to the practitioner during clinical encounters). In ‘asymmetric encounters’ cases, decisional needs concerned ‘prioritization’ (*n =* 10), ‘use of services’ (*n* = 3) and ‘prescription’ (*n* = 8) decisions. Of 21 cases, 16 presented a lack of patient-practitioner communication and seven interprofessional coordination issues.

In this decision-making configuration, all 21 cases presented non-informed decisions, or decisions that were not based on patients’ values. Eighteen reported action-related issues (e.g., non-adherence, delaying the decision), and 18 reported negative well-being impacts (e.g., patients felt frustrated or pressured). In addition, 20 cases reported negative outcomes and one case presented an equivocal outcome, which related to antibiotics and steroids prescriptions for patients with Chronic Obstructive Pulmonary Disease (COPD). In the ‘asymmetric encounters’ configuration, no case reported using a decision support tool.

#### Self-management by default

In this configuration, eight cases grouped into two patterns with similar conditions (Fig. 3). Decisions were made by PCCNs and their caregivers independently, and between clinical encounters. ‘Self-management by default’ cases concerned ‘prioritization’ (*n =* 3) and ‘use of services’ (*n =* 5) decisional needs. Of these eight cases, seven reported negative outcomes, and one an equivocal outcome. The negative outcomes corresponded to patients who felt overwhelmed and abandoned in the self-management of their multimorbidity and experienced polypharmacy. Some patients explained that their day-to-day routine required a great deal of energy and time due to their multiple health and social issues. The eight cases reported non-informed based decision related communicational issues. One case presented an inappropriate underuse of services and delays in a decision leading to hospitalization (action issues). Two cases reported caregivers’ cognitive burden when the patient decided to refuse services. Two other cases presented patients/caregivers in situation of social vulnerability that affected self-management. In the case with an equivocal outcome, the patients were guided by their values and actively engaged in their care, but they decided not to adhere to recommended prioritization or services while they had not been adequately informed of the consequence of their action. No case reported a decision-making tool.

#### Chaotic

In this configuration, 27 cases with similar conditions were grouped together into one pattern (the red square in Fig. 3). In this decision-making configuration, all cases had negative outcomes associated with mental health and social issues, and unsatisfactory patient-practitioner communication and interprofessional coordination (e.g., between primary care services and other services, specifically mental health and social care services). All decisions were made independently by PCCNs, their caregivers and practitioners (between clinical encounters). The ‘chaotic’ decision-making configuration concerned all types of decisional needs: ‘use of services’ (*n =* 10), ‘institutionalization’ (*n =* 5), ‘behavioural change’ (*n =* 4), ‘prescription’ (*n =* 4), and ‘prioritization’ (*n =* 4). Almost all cases concerned patients in situation of social vulnerability (*n =* 26), often with high disability, e.g., frailty with dementia (*n =* 18). Some patients were homeless, or experienced financial precariousness (*n =* 8). In addition, many cases presented patients facing mental health issues (*n =* 19). Twenty-five cases reported action-related issues (e.g., patient non-engagement, delay of decision, access to services, and non-adherence). Emotional impacts of decisions were negative in 21 cases: frustration, loneliness, regret, and uncertainty about the decision made, and feeling pressured. Fifteen cases reported disagreements among stakeholders (caregivers, patients, and practitioners). Some patients (e.g., marginalized people), expressed a loss of trust in the health system. In this configuration, those making decisions involving behavioural change or institutionalization reported major family or cultural conflicts (*n =* 9). This configuration also concerned cases reporting a refusal of social services (*n =* 4), consultation and navigation issues in a fragmented health system (*n =* 3), non-adherence to prescriptions by patients in situations of social vulnerability (*n =* 3), marginalized patients prioritizing urgent problems with their daily living activities over health care (*n =* 3), and frequent hospitalization of socially isolated frail elders (*n =* 2). No case reported a PCCN-oriented tool.

## Discussion

This systematic review contributes to the ongoing reflections on decision-making by considering the challenging social contexts underlying the decisional needs among PCCNs. It includes 47 empirical studies from 13 countries about PCCNs in primary care, representing a large sample of 2997 participants (2107 patients, 698 practitioners and 192 caregivers). The case-based qualitative synthesis allowed us to identify 69 cases of decisional needs within the 47 studies. The five main types of decisional needs among PCCNs concerned ‘prioritization’, ‘use of services’, ‘prescription’, ‘behavior change’ and ‘institutionalization’. For each case, we wrote a ‘6 W-O’ memo and synthesized data, which generated four decision-making configurations in the form of spider web diagrams, illustrating patterns of conditions associated with decision outcomes. We entitled the four decision-making configurations ‘well-managed’, ‘asymmetric encounters’, ‘self-management by default’, and ‘chaotic’. These configurations provide explanations and testable predictions, an endeavour of some urgency given the growing proportion of older adults and rising rates of chronic disease [2, 82]. Our results suggest the following nine main contributions.

### Five types of decisional needs

#### Prioritization: difficulty prioritizing which multiple mental, physical, and social issues to address is common among PCCNs

The most common PCCNs’ decisional need was a difficulty prioritizing which mental, physical, or social issue to address. Some ‘prioritization’ cases illustrate that before sharing information on care options, stakeholders need to decide which information is most relevant to address. Prioritization tools and strategies exist for PCCNs [73], and their use may lead to positive outcomes (i.e., the ‘well-managed’ configuration). However, all independent prioritization cases, i.e., when practitioners or patients prioritize alone, lead to negative outcomes (e.g., the ‘chaotic’ configuration). Our results support the literature in finding that prioritization is a challenge for the PCCNs for four main reasons. Firstly, their experience of multimorbidity and polypharmacy increases tenfold the information to be communicated during consultations [42, 56]. Second, these decisions are rarely shared between elderly PCCNs and their practitioners and sharing them is currently perceived as unusual by both parties [49]. Third, PCCNs are more likely to suddenly change their decision about what issue to prioritize because of a cascade of crises or sudden deterioration in their own health [69]. Fourth, PCCNs in situations of social vulnerability (socio-economically deprived, highly disabled and/or marginalized people) often have no choice but to prioritize daily living activities over health issues [50, 62, 63]. This can lead to underuse of services needed, in turn leading to use of emergency services and hospitalization when a crisis point is reached, and even less likelihood they will share decisions with their healthcare provider.

#### Use of services: frequent users and under users of services are in situations of social vulnerability

We found ‘use of services’ type decisional needs across all four decision-making configurations, but it was particularly common in the ‘self-management by default’ and ‘chaotic’ configurations, in which all outcomes were negative. ‘Use of services’ is a common type of decisional need among frequent users [17]. In fact, this type of decisional needs included the highest proportion of frequent users of services, who are seen as difficult patients by some practitioners [55]. These patients are often people with disabilities, e.g., frail and elderly [64]. In addition, those with the ‘use of services’ type of decisional need included the highest proportion of under users of service in relation to their needs. These are mostly marginalized people [50].

#### Prescription: PCCN may be confronted to poor communication with their prescribers and lack of interprofessional coordination

The ‘prescription’ type of decisional needs is mainly concerned patients with multimorbidity and polypharmacy. In most cases of PCCNs ‘prescription’ decisional needs, the patient plays a passive role in the treatment decision-making process vis-a-vis the practitioner. Prescription or deprescription decisions are mostly taken independently by practitioners. Some cases concerned non-adherence to prescription decision made by the patients independently between clinical encounters. Some patients in situations of polypharmacy stopped or reduced medication because they were afraid of iatrogenic drug-to-drug interaction [75]. They criticized the poor patient-practitioner communication and lack of interprofessional coordination between too many prescribers. Cases of non-adherence to medication could also result from a tacit prioritization process [59]: most of these cases were in situations of socio-economic deprivation, so the decision to not adhere could be interpreted as a result of inability to pay, or a choice to prioritize daily living activities over medication.

#### Behavior change: changing lifestyle habits can be challenging in the context of social vulnerabilities

Changing behavior is a difficult decision for some patients in a situation of social vulnerability who chose independently to stop or continue an unhealthy habit (e.g., smoking). All cases in this decisional need type are mapped on to the ‘chaotic’ configuration and reported a lack of communication between practitioners and highly disabled patients in socio-economic deprivation, as also found in studies on COPD [55]. The adoption of new lifestyle habits and behaviors is a major challenge in case management programs [17].

#### Institutionalization: caregivers experienced individual, interactional and organizational issues

‘Institutionalization’ is the type of decisional needs with the highest level of complexity, following Luhmann’s definition, i.e., that it repeatedly puts pressure on stakeholders to select an option despite their uncertainties about the options in terms of health and social services. Typically, overwhelmed caregivers must decide for PCCNs, e.g., elderly patients with dementia, when the patients’ behavior becomes unmanageable. Advocating the patient’s perspective is however sometimes difficult for caregivers who are faced with having to institutionalize a loved one (due to unmanageable critical situations at home) even against his or her wishes [44]. These cases are often experienced as heartbreaking by caregivers. They felt alone in making the decision. No case reported that caregivers were supported by a decision-making tool or program. The delay before institutionalization occurred also had negative impacts on caregivers, who had to investigate and approach several different institutions [37]. Moreover, all ‘institutionalization’ cases involved a family conflict that was sometimes aggravated by generational cultural conflicts and decision-makers taking positions as either allies or competitors [65].

### Four decision-making configurations

#### Well-managed: PCCNs’ decisions made in partnership are associated with positive outcomes

By comparing the ‘well-managed’ cases with those in the three other decision-making configurations, we observed that decisions made in partnership were associated with positive outcomes. In this configuration, decision quality seemed informed and value-based. This configuration contains 13 cases sharing similar outcomes and conditions; it suggests a testable prediction for future research (Table 3).

**Table 3:** Well-managed configuration testable prediction

Regarding PCCNs’ decisional needs of the ‘prioritization’ and ‘use of services’ types, positive outcomes seem more likely when three conditions are met: (a) sufficient information is shared, understood and acted upon by PCCNs, their caregivers and all involved practitioners [satisfactory patient-practitioner communication and coordination between practitioners in primary care and other health and social care services], (b) all inter-related decision-making processes are based on PCCNs’ and caregivers’ trust in their partnership with practitioners during every clinical encounter, and are centered on PCCN’s values and preferences, and (c) appropriate PCCN-oriented tools, strategies, or programs are used to reduce the complexity of the inter-related decision-making processes, and care needs.	

#### Asymmetric encounters: informational and power asymmetries between PCCNs and practitioners

This decision-making configuration exposed informational and power asymmetries between PCCNs and practitioners during clinical encounters. All decisions in this configuration were made independently by practitioners or by patient during clinical encounters, resulting in non-informed or non-value based decisions about ‘prioritization’, ‘use of services’ or ‘prescription’. Practitioners make independent decisions because they only consider medical explanations of disease, in isolation from the PCCNs’ own perceptions of their illness and their knowledge of its consequences on their lives [83, 84]. On the other hand, patients also intentionally omit certain information. This voluntary omission could be interpreted as an act of resistance to power asymmetry [85, 86]. Some informational asymmetries can be explained by an unconscious ‘clinical habitus’ [87], socially embodied during patients’ experiences in other clinical settings, e.g., to adopt passive roles during clinical encounters and what information is pertinent to share with the practitioner [88, 89].

#### Self-management by default: PCCNs felt overwhelmed and abandoned

The ‘self-management by default’ decision-making configuration illustrates that many PCCNs’ and caregivers’ decisions are made between clinical encounters. This configuration suggests that decisions made independently by PCCNs between clinical encounters lead to negative outcomes (e.g., patient or caregiver burden). It highlights the importance of better decision support for the self-management of PCCNs who feel overwhelmed by situations of multimorbidity and social vulnerability. The PCCNs self-managed their decisions by default because they did not have access to decisional support. The ‘self-management by default’ and the ‘asymmetric encounters’ configurations share similar outcomes and conditions (except for where the decision takes place, i.e., during or between), and together they suggest the following testable predictions for future research (Table 4).

**Table 4:** Self-management by default and asymmetric encounters configurations testable prediction

With respect to decisional needs relating to ‘prioritization’, ‘use of services’ and ‘prescription’, negative outcomes (including poor health outcomes and caregiver burden) seem more likely when decisions are made independently by patients/caregivers or practitioners (between or during clinical encounters). The following conditions seem to contribute to negative outcomes: PCCNs’ mental health issues or social vulnerability, unsatisfactory patient-practitioner communication or interprofessional coordination, and non-use of appropriate PCCN-oriented tools, strategies, or programs (no practical attempt to decrease complexity).	

#### Chaotic: decisions in times of crisis or mistrust services

The ‘chaotic’ configuration included the largest number of cases and involved all types of decisional needs. This decision-making configuration illustrates that there are decision situations that are mostly beyond the scope of any decision support tools. It seems that only high intensity health and social care programs, e.g., high quality integrated multidisciplinary care teams, can prevent the worsening of outcomes. Typically, PCCNs in this decision-making configuration were in situations of extreme social vulnerability (socio-economic deprivation, highly disabled and/or marginalized). According to our knowledge users with expertise in social work, the ‘chaotic’ configuration reminds us that some marginalized people are not in a moment in time or in a state of mind to be able to accept services. It emphasizes the importance of the principle of Kairos, a dimension of time qualified as an opportune moment. For example, a social worker must intuitively sense when the time is right to reach people who mistrust services and see themselves excluded from the system. This configuration included 27 cases sharing similar outcomes and conditions, suggesting a testable prediction for future research (Table 5).

**Table 5:** Chaotic configuration testable prediction

Across all types of PCCNs decisional needs, negative outcomes are more likely when four conditions are met: (a) patients face a crisis, are not reachable, fall through the cracks of the fragmented health and social care systems, and/or are unable to navigate its complexities; (b) patient-practitioner communication and interprofessional coordination are unsatisfactory; (c) decisions are made independently by patients between clinical encounters because they mistrust services, i.e., patients perceive themselves as excluded from the health and social care systems (and other systems) and practitioners see these patients as an unwanted burden; and (d) no PCCN-oriented high intensity program is applied or applicable.	

### Theorical contributions: toward a PCCNs-adapted decision-making model

The decisional needs typology and the decision-making configurations can contribute to a PCCNs-adapted decision-making model. The shared decision-making model (SDM) advocates for a process where patient and practitioner work together to make an informed choice congruent with patient’s values (see additional file 5). According to Charles et al. [90, 91], information sharing is a prerequisite to SDM. However, the ‘prioritization’ type of decisional need indicates that before sharing information, PCCNs and their practitioners need to decide which information to address in priority. Adaptation of SDM for PCCNs should consider that this prioritization process can be challenging in this context due to the large amount of information and goals of care to be prioritized. In addition, people in situations of social vulnerability may have to prioritize daily living activities over health issues. The decisional needs type identified in this study show that PCCN face many more decisions than simply choosing a treatment option. Furthermore, the results highlight the importance of considering social vulnerabilities in PCCNs’ decision-making. This consideration is particularly important for understanding lifestyle habits or services use, which is sometimes deemed inappropriate by some practitioners (e.g., their perception of an overuse of services).

As Charles et al. [90] suggest, the complexity of interactional dynamics increases when agents with different perspectives interact in the decision-making processes. This situation is obvious in cases of ‘institutionalization’ where caregivers must take heartbreaking decisions in the context of family conflicts that can be aggravated by generational cultural conflicts [65]. As Charles et al. [92] have pointed out, few studies in the treatment decision-making field were culturally sensitive. The development of a culturally sensitive caregiver-as-agent model and tools to support caregivers confronted with individual, interactional, and organizational issues are needed [43].

The ‘well-managed’ configuration takes into account situations where it would be possible to apply SDM with PCCNs. Indeed, this configuration-related decision process seems congruent with the key SDM characteristics suggested by Charles et al. [90] and is associated with positive outcomes. On the contrary, the three other configurations show situations where SDM is difficult, and are associated with negative outcomes. The ‘asymmetric encounters’ configuration exposes informational and power asymmetries between PCCNs and practitioners during clinical encounters, a barrier to overcome for applying SDM. The ‘self-management by default’ and the ‘chaotic’ configurations reminds us that many decisions take place outside the context of the medical encounter, a limit to the SDM recognized by Charles et al.’s [91]. In comparison to the ‘chaotic’ configuration, an encouraging element of the ‘self-management by default’ is that PCCNs remained reachable and have the cognitive capacities to participate in decision-making processes and might consider a PCCN-oriented decision-making tool. The ‘chaotic’ configuration considers situations beyond the scope of SDM, which is inapplicable in times of crisis or mistrust services.

### Practical implications

Findings from qualitative syntheses can help guide policy and practice in many disciplines [93]. Our results show no tool or strategy linked to three non-well managed decision-making configurations (asymmetric encounters, self-management by default, chaotic), which in turn suggest a need for further research and development. In addition, they present PCCN-oriented tools, strategies and programs that are associated with the ‘well-managed’ configuration. This suggests that we must maintain the development and implementation of interventions and tools for all types of decisional needs.

In the literature, several services and tools for the diagnosis and management of complex care needs have been identified. For example, systematic reviews have shown that case management can improve PCCNs satisfaction, self-management, quality of care, services use/costs, and health outcomes [94–97]. Decision aids have recently been proposed to improve patient engagement in case management programs [98]. In addition, tools exist that aim to detect, assess, prioritize, and follow up complex care needs, and ultimately to improve PCCN/caregiver-practitioner communication, and communication between practitioners. For example, the *Comprehensive Geriatric Assessment Toolkit +* (cgakit.com) maps 80 validated tools for needs assessment and prioritization. Typically, tools can be completed during or between clinical encounters, usually by practitioners, rarely by PCCNs and their caregivers, or both (practitioners and PCCNs and their caregivers). They can provide useful information to patients facing complex situations. The main obstacle is lack of awareness of the tools (most are unknown to practitioners, PCCNs and their caregivers) and lack of integration of these tools into practitioners’ workflow and PCCNs’ and caregivers’ routines.

Because complex care needs often involve multiple stakeholders, it is essential to broaden the conceptualization of SDM beyond the physician-patient dyad [99]. The Inter-Professional Shared Decision-Making (IP-SDM) model extends SDM to include family members and other caregivers as well as a team of professionals in a patient-centered process [100]. This model aims to stimulate deliberation and reaching a common understanding among all parties involved in the decision. PCCNs, whose decisional needs often involve a multitude of parties, may greatly benefit from IP-SDM programs. For example, the IP-SDM Dolce (Decision-making On Location of Care with the frail Elderly and their Caregivers) focused on the role of caregivers in the tough decisions about the institutionalization of their loved ones [101]. If it is important to consider the decisional needs of PCCNs, we must consider the decision-making needs of caregivers who often feel overwhelmed by difficult decisions, especially when they are linked to family conflict. The Family Caregivers Support Agreement, a tool proposed in one of the studies identified in our review, offers a support to consider the needs of caregivers in decision-making [43].

### Limitations and strengths

Our case-based qualitative synthesis has five main limitations. First, only detected PCCNs were involved in included studies, while PCCNs are usually under-detected [82, 102]. The number of cases with negative outcomes was higher than those with positive outcomes, which may suggest that PCCNs facing severe issues were overrepresented in the included studies. Also, as with all systematic reviews, due to publication bias, this work may be biased by predetermined outcomes that were identified by authors of the included studies. However, this limitation was somewhat reduced by the knowledge users in our study (patient-partner, practitioners, service-managers, and policy-makers), who discussed and approved the case-based synthesis of the data extracted from included studies. Five of them who had relevant expertise validated the cases. Second, we defined complexity of care needs in accordance with Luhmann’s theory, which assumes that PCCNs and caregivers are integrated into social systems [10], while competing social theories suggest otherwise. Third, because of the specificity of their decisional needs, we excluded two PCCN populations: children with complex care needs and people at the end of life (palliative care). Fourth, we classified cases by types of decisional needs, but patient’s decisional needs may evolve and change over time for a variety of reasons (a cascade of crises, self-perceptions of control, clinician interactions). The outcomes of a decision can also have an impact on other decisions. Fifth, the search strategy was conducted until December 2017. However, this systematic mixed studies review proposes an innovative, empirically validated, and testable conceptual framework of decisional needs among PCCNs, and outcomes related to decision. This conceptual framework is original. We reviewed the literature published since completing our search strategy (Scopus database search up to October 2021) and found no framework or theory of decisional needs among PCCNs and outcomes related to decision. Following data sufficiency criterion [103, 104] our sample provided ample qualitative and quantitative data to compare constructs and dimensions, reflect the work of several independent researchers, and reach our objectives. Compared to usual qualitative syntheses, we included a large number of studies (47 empirical studies) and we identified 69 decisional needs cases representing 2997 participants from 13 countries.

Several other strengths of our review should be underscored. Knowledge user involvement in this review helped to understand decision-making processes in complex care needs situations and help to build a conceptual framework based on many cases [105]. Our synthesis was case-based, which helps generate innovative, empirically validated, and testable conceptual frameworks [93, 105]. While there is no ideal number of cases to build a theory, Eisenhardt [106], an expert in the field, suggested a minimum of four cases. This review offers rich theoretical, methodological, and practical contributions. Luhmann’s conception of complexity is broader and more comprehensive than is included in usual definitions of complex care needs. Combining this with the Charles et al.’s decision-making model [90–92, 107, 108] and the Ottawa Decision Support Framework [16] led to a comprehensive description of PCCNs’ decisional needs. There was a large sample of participants enrolled in the included studies, which allowed us to cover a diversity of PCCNs-related contexts. This diversity enabled us to extend the Charles et al.’s model with five types of decisional needs prevalent among PCCNs and four decision-making configurations. Three testable predictions are proposed for future research (see Tables 3, 4 and 5). This case-based qualitative synthesis helped bridge two knowledge gaps: (a) the majority of intervention studies address simple care needs rather than complex ones; and (b) current systematic reviews typically focus on one health condition and one homogeneous population [109, 110].

## Conclusions

The ultimate contribution of this review is a typology of PCCNs’ decisional needs and testable predictive decision-making configurations. The decisional needs typology shows that in contexts of social vulnerability many health decisions are made outside clinical encounters and concern issues other than treatment options. The decision-making configurations show that shared decision-making is associated with positive outcomes, and that negative outcomes are associated with practitioners, patients or caregivers making health decisions independently. This can contribute to informing the subsequent user-centered design of decision support tools.

## Supplementary Information

**Additional file 1.** PRISMA 2009 Checklist and the ENTREQ statement.

**Additional file 2.** Search Strategy.

**Additional file 3.** Decisional needs cases description.

**Additional file 4.** Quality appraisal of the 47 included studies.

**Additional file 5.** Treatment decision-making models.

## Data Availability

The dataset supporting the conclusions of this article is included within the article and its additional files.

## Abbreviations

6 W-O: What, Who, How, Where, When, Why and related Outcomes
MMAT: Mixed Methods Appraisal Tool
ODSF: Ottawa Decision Support Framework
PCCNs: Patients with Complex Care Needs
SDM: Shared Decision-Making
