# Supplementary material for: Decision-making and related outcomes of patients with complex care needs in primary care settings: a systematic literature review with a case-based qualitative synthesis
Source: BMC Prim Care. 2022 Nov 9;23:279. doi: 10.1186/s12875-022-01879-5 (PMC9644584; doi:10.1186/s12875-022-01879-5)
Supplement: Supplementary file 1 — Additional file 1. PRISMA 2009 Checklist and the ENTREQ statement. [file 12875_2022_1879_MOESM1_ESM.docx]

**Additional file [1](https://bmcfampract.biomedcentral.com/articles/10.1186/s12875-020-01107-y#MOESM1) – PRISMA checklist and ENTREQ statement**

**PRISMA 2009 Checklist and**

| **Section/topic** | **#** | **Checklist item** | **Reported on page #** |
| --- | --- | --- | --- |
| **TITLE** | | |  |
| Title | 1 | Identify the report as a systematic review, meta-analysis, or both. | p.1 |
| **ABSTRACT** | | |  |
| Structured summary | 2 | Provide a structured summary including, as applicable: background; objectives; data sources; study eligibility criteria, participants, and interventions; study appraisal and synthesis methods; results; limitations; conclusions and implications of key findings; systematic review registration number. | p.1-2 |
| **INTRODUCTION** | | |  |
| Rationale | 3 | Describe the rationale for the review in the context of what is already known. | p.2-4 |
| Objectives | 4 | Provide an explicit statement of questions being addressed with reference to participants, interventions, comparisons, outcomes, and study design (PICOS). | p.4 |
| **METHODS** | | |  |
| Protocol and registration | 5 | Indicate if a review protocol exists, if and where it can be accessed (e.g., Web address), and, if available, provide registration information including registration number. | p.4 |
| Eligibility criteria | 6 | Specify study characteristics (e.g., PICOS, length of follow-up) and report characteristics (e.g., years considered, language, publication status) used as criteria for eligibility, giving rationale. | p.5 |
| Information sources | 7 | Describe all information sources (e.g., databases with dates of coverage, contact with study authors to identify additional studies) in the search and date last searched. | p.5 |
| Search | 8 | Present full electronic search strategy for at least one database, including any limits used, such that it could be repeated. | p.5 |
| Study selection | 9 | State the process for selecting studies (i.e., screening, eligibility, included in systematic review, and, if applicable, included in the meta-analysis). | P.5-6 |
| Data collection process | 10 | Describe method of data extraction from reports (e.g., piloted forms, independently, in duplicate) and any processes for obtaining and confirming data from investigators. | p.5-6 |
| Data items | 11 | List and define all variables for which data were sought (e.g., PICOS, funding sources) and any assumptions and simplifications made. | p.6-9 |
| Risk of bias in individual studies | 12 | Describe methods used for assessing risk of bias of individual studies (including specification of whether this was done at the study or outcome level), and how this information is to be used in any data synthesis. | p.6 and 11 |
| Summary measures | 13 | State the principal summary measures (e.g., risk ratio, difference in means). | N/A |
| Synthesis of results | 14 | Describe the methods of handling data and combining results of studies, if done, including measures of consistency (e.g., I^2^) for each meta-analysis. | p.6-9 |
| Risk of bias across studies | 15 | Specify any assessment of risk of bias that may affect the cumulative evidence (e.g., publication bias, selective reporting within studies). | p.32 |
| Additional analyses | 16 | Describe methods of additional analyses (e.g., sensitivity or subgroup analyses, meta-regression), if done, indicating which were pre-specified. | N/A |
| **RESULTS** |  |  |  |
| Study selection | 17 | Give numbers of studies screened, assessed for eligibility, and included in the review, with reasons for exclusions at each stage, ideally with a flow diagram. | p.10 |
| Study characteristics | 18 | For each study, present characteristics for which data were extracted (e.g., study size, PICOS, follow-up period) and provide the citations. | p.10-11 |
| Risk of bias within studies | 19 | Present data on risk of bias of each study and, if available, any outcome level assessment (see item 12). | p.11 and Additional file 3 |
| Results of individual studies | 20 | For all outcomes considered (benefits or harms), present, for each study: (a) simple summary data for each intervention group (b) effect estimates and confidence intervals, ideally with a forest plot. | Additional file 3 |
| Synthesis of results | 21 | Present results of each meta-analysis done, including confidence intervals and measures of consistency. | p.12-23 |
| Risk of bias across studies | 22 | Present results of any assessment of risk of bias across studies (see Item 15). | p.11 |
| Additional analysis | 23 | Give results of additional analyses, if done (e.g., sensitivity or subgroup analyses, meta-regression [see Item 16]). | N/A |
| **DISCUSSION** |  |  |  |
| Summary of evidence | 24 | Summarize the main findings including the strength of evidence for each main outcome; consider their relevance to key groups (e.g., healthcare providers, users, and policy makers). | p.23-31 |
| Limitations | 25 | Discuss limitations at study and outcome level (e.g., risk of bias), and at review-level (e.g., incomplete retrieval of identified research, reporting bias). | p.32-34 |
| Conclusions | 26 | Provide a general interpretation of the results in the context of other evidence, and implications for future research. | p.34 |
| **FUNDING** |  |  |  |
| Funding | 27 | Describe sources of funding for the systematic review and other support (e.g., supply of data); role of funders for the systematic review. | p.40 |

**Elements of the ENTREQ statement addressed in the manuscript** (adapted from Tong et al. 2012, see table 1, p 4)

| **Item Guide** | **Description** | **Addressed** |
| --- | --- | --- |
| **Aim** | State the research question the synthesis addresses. | X |
| **Synthesis methodology** | Identify the synthesis methodology or theoretical framework which underpins the synthesis, and describe the rationale for choice of methodology. | X |
| **Approach to searching** | Indicate whether the search was pre-planned (comprehensive search strategies to seek all available studies) or iterative (to seek all available concepts until they theoretical saturation is achieved). | X |
| **Inclusion criteria** | Specify the inclusion/exclusion criteria (e.g. in terms of population, language, year limits, type of publication, study type). | X |
| **Data sources** | Describe the information sources used. | X |
| **Electronic Search strategy** | Describe the literature search. | X |
| **Study screening methods** | Describe the process of study screening and sifting. | X |
| **Study characteristics** | Present the characteristics of the included studies. | X |
| **Study selection results** | Identify the number of studies screened and provide reasons for study exclusion. | X |
| **Rationale for appraisal** | Describe the rationale and approach used to appraise the included studies. | X |
| **Appraisal items** | State the tools, frameworks and criteria used to appraise the studies or selected findings. | X |
| **Appraisal process** | Indicate whether the appraisal was conducted independently by more than one reviewer and if consensus was required. | X |
| **Appraisal results** | Present results of the quality assessment and indicate which articles. | X |
| **Data extraction** | Indicate which sections of the primary studies were analysed and how were the data extracted from the primary studies? | X |
| **Software** | State the computer software used, if any. | X |
| **Number of reviewers** | Identify who was involved in coding and analysis. | X |
| **Coding** | Describe the process for coding of data. | X |
| **Study comparison** | Describe how were comparisons made within and across studies. | X |
| **Derivation of themes** | Explain whether the process of deriving the themes or constructs was inductive or deductive. | X |
| **Quotations** | Provide quotations from the primary studies to illustrate themes/constructs, and identify whether the quotations were participant quotations of the author’s interpretation. |  |
| **Synthesis output** | Present rich, compelling and useful results that go beyond a summary of the primary studies (e.g. new interpretation, models of evidence, conceptual models, analytical framework, development of a new theory or construct). | X |

Tong, A., Flemming, K., McInnes, E., Oliver, S., & Craig, J. (2012). Enhancing transparency in reporting the synthesis of qualitative research: ENTREQ. *BMC Med Res Methodol, 12*, 181. doi:10.1186/1471-2288-12-181
