# Supplementary material for: Decision-making and related outcomes of patients with complex care needs in primary care settings: a systematic literature review with a case-based qualitative synthesis
Source: BMC Prim Care. 2022 Nov 9;23:279. doi: 10.1186/s12875-022-01879-5 (PMC9644584; doi:10.1186/s12875-022-01879-5)
Supplement: Supplementary file 2 — Additional file 2. Search Strategy. [file 12875_2022_1879_MOESM2_ESM.docx]

**Additional file 2 –** **Search Strategy**

| 1 (complex adj4 (problem* or issue* or patient? or need? or care or existence? or experience? or live? or realit* or journey? or situation?)).ti,ab,kf. |
| --- |
| 2 complex case?.mp. |
| 3 (complexity adj4 (clinical or patient? or science or theory)).mp. |
| 4 ((high-effort or burden or complicated or demanding) adj patient?).mp. |
| 5 exp Vulnerable Populations/ |
| 6 poverty/ or poverty areas/ or unemployment/ or homeless persons/ or homeless youth/ or exp *aged/ or frail elderly/ or exp "Emigrants and Immigrants"/ or minority groups/ or exp disabled persons/ or drug users/ or medically uninsured/ or refugees/ or exp culture/ |
| 7 (poverty or disadvantaged or underserved or under served or indigen* or tribe? or tribal or native? or aboriginal* or low income* or unemploy* or underemploy* or under employ* or homeless* or street people or street person? or (social* adj (isolat* or stigma*)) or inequalit* or uninsured or underinsured or unader insured or uneducated or low* educat* or poor* educat* or illitera* or (low adj2 litera*) or functional* impair* or disabled or disabilit* or handicap* or physical* challenge* or mental* challenge* or ((drug or substance) adj (abuse* or addict* or dependen* or habit? or "use*")) or minorit* or emigra* or immigra* or migra* or foreigner* or refugee*).ti,ab,kf. |
| 8 (vulnerab* or aged or elderly or frail* or senior?).ti. |
| 9 ((frail* or vulnerab* or at risk or high risk or low function or dependent) adj2 (older or elder* or senior* or patient*)).ti,ab,kf. |
| 10 (cald or (cultural* adj3 divers*) or multicultur* or intercultur* or (patient* adj cultur*) or (cultural* adj3 (background* or differen*)) or ethnocultural* or (cultural* adj (aware* or competen* or appropriate* or relevan* or safe* or train*))).ti,ab,kf. |
| 11 (vulnerab* adj (patient? or population? or social*)).ti,ab,kf. |
| 12 sensitive population?.ti,ab,kf. |
| 13 ((Frequen* or high) adj2 (attend* or consult*)).ti,ab,kf. |
| 14 ("frequent visit*" or "frequent flyer*" or "heavy user*" or "repeat use").ti,ab,kf. |
| 15 ((((frequen* or high) adj2 (user* or utili*)) or "high use" or "frequent use") adj3 (patient* or hospital* or emergency or ED or services)).ti,ab,kf. |
| 16 "revolving door".ti,ab,kf. |
| 17 "frequent hospitali#ation*".ti,ab,kf. |
| 18 ((preventable or avoidable) adj2 (utili* or visit* or hospitali* or consultation*)).ti,ab,kf. |
| 19 (high adj2 risk adj3 hospitali#ation*).ti,ab,kf. |
| 20 ("frequent use*" or "frequent utilis*" or "high use*" or "high utili*").kf. |
| 21 mental disorders/ or mental health/ |
| 22 ((mental* or psychiatric) adj (health* or disorder* or disease* or ill*)).ti. |
| 23 comorbidity/ |
| 24 (comorbidit* or multi* morbidit* or multimorbidit*).ti,ab,kf. |
| 25 exp polypharmacy/ |
| 26 exp drug interactions/ |
| 27 exp "Drug-Related Side Effects and Adverse Reactions"/ |
| 28 (adverse adj (effect? or event? or reaction?)).ti. |
| 29 ((multi* adj (therap* or treatment* or drug? or medication?)) or polypharmac*).ti,ab,kf. |
| 30 drug* interact*.ti,ab,kf. |
| 31 exp complementary therapies/ |
| 32 exp herbal medicine/ |
| 33 ((alternative* or complementar* or folk* or herbal or integrat* or natural or non-prescription or over the counter or traditional) adj2 (health* or medication* or medicine* or product* or remedy or remedies or therap* or treatment*)).ti,ab,kf. |
| 34 or/1-33 |
| 35 exp Primary Health Care/ |
| 36 exp Primary Care Nursing/ |
| 37 exp General Practice/ |
| 38 Community Health Services/ |
| 39 exp Community Pharmacy Services/ |
| 40 Community Mental Health Services/ |
| 41 Community Health Nursing/ |
| 42 Social Work/ |
| 43 General Practitioners/ |
| 44 Physicians, Family/ |
| 45 Physicians, Primary Care/ |
| 46 Social Workers/ |
| 47 (primary care or primary health care or primary healthcare or community nursing or family practice or general practice or family medicine or family physician* or family practitioner* or family doctor* or general physician* or general practitioner* or community based medicine or community mental health service* or community mental health nursing or community health nursing or community health service* or community pharmac* or primary practice or primary practitioner* or psychologist* or social service* or social work* or (communit$3 adj5 nurse?)).ti,ab,kf. |
| 48 or/35-47 |
| 49 exp Interpersonal Relations/ |
| 50 exp patient care team/ |
| 51 (exp nurses/ or exp physicians/ or pharmacists/ or social workers/ or (nurse* or pharmacist* or physician* or psychologist* or social worker* or clinician* or doctor* or practitioner* or gps or health care professional* or healthcare professional* or health care provider* or healthcare provider* or ((primary care or primary healthcare or primary health care) adj provider*) or resident*).ti.) and (exp patients/ or caregivers/ or exp Family/ or (patient* or consumer* or people* or carer? or caregiver? or family or families).ti.) |
| 52 exp consumer participation/ or ((patient* or consumer*) adj6 (interaction* or empower* or engagement* or involvement* or involving* or participation* or participating*)).ti,ab,kf. |
| 53 (exp patients/ or (patient* or inpatient* or outpatient* or hospitali#ed or institutionali#ed or consumer* or people*).ti.) and (caregivers/ or exp Family/ or (carer* or caregiver* or family or families).ti.) |
| 54 (collaborat* or team*).ti,ab,kf. |
| 55 (interprofessional* or inter professional* or interdisciplinar* or inter disciplin* or interoccupation* or inter occupation* or multiprofessional* or multi professional* or multidisciplin* or multi disciplin* or multioccupation* or multi occupation*).ti,ab,kf. |
| 56 (interpersonal* or shared care).ti,ab,kf. |
| 57 or/49-56 |
| 58 (decision* or decided or decides or deciding or choice*).ti,ab,kf. |
| 59 exp decision making/ or informed consent/ or exp problem solving/ or (exp patient preference/ and patient education as topic/) |
| 60 ((patient* adj3 (voice* or perspective*)) or preference* or deliberation* or navigat* or accommodation* or accord? or agree* or arrangement or compromise or conciliation or counterbalance or counterpoise or equipoise or mediation or negotia* or poise or prioriti?ation or prioriti?e* or prioriti?ing or reconciliation).ti,ab,kf. |
| 61 (regret* or blame* or uncertaint* or disagreement or disconcerted or faithless or dissension or dissent* or distrust* or indecision or indecisive or refusal or trustless or undecided or untrustworthy or untrusting or mistrust*).ti,ab,kf. |
| 62 or/58-61 |
| 63 34 and 48 and 57 and 62 |
| 64 Limit 63 to (English or French or Spanish) |
