# Supplementary material for: Decision-making and related outcomes of patients with complex care needs in primary care settings: a systematic literature review with a case-based qualitative synthesis
Source: BMC Prim Care. 2022 Nov 9;23:279. doi: 10.1186/s12875-022-01879-5 (PMC9644584; doi:10.1186/s12875-022-01879-5)
Supplement: Supplementary file 3 — Additional file 3. Decisional needs cases description. [file 12875_2022_1879_MOESM3_ESM.docx]

# Additional file 3 Table - Decisional needs cases description

**Acronyms:** CC (Cultural conflict); FC (family conflict); FU (Frequent Users); IP (Interprofessional coordination issues); LC (lack of patient-practitioner communication); MH (Mental Health issues); MM (Multimorbidity); P (Program); PP (Polypharmacy); S (Strategy); SV (Social Vulnerability); SV1 (socio-economic deprivation); SV2 (highly disabled people, e.g., frail elderly); SV3 (marginal, e.g., homeless); T (Tool); UU (Under Users).

## 3.1 Prioritization made independently during clinical encounters

| **Case ID** | **Studies** | **What** (decision) | | **Outcomes**  (quality, actions, impacts) | **Who** (PCCN characteristics) | **Why** (Interactional conditions) | **How** (Independent / partnership) | **Tool or strategy** (to facilitate decision) | **Decision-making configuration** |
| --- | --- | --- | --- | --- | --- | --- | --- | --- | --- |
| 295.1 | Paskins 2015 | | Identifying health and psychosocial issues | Negative: quality, actions, impact | MM | LC | Independent (Practitioner) | no | Asymmetric  encounter |
| 295.2 | Paskins 2015 | | Prioritizing health and psychosocial issues | Negative: quality, impact  Cannot tell: actions | MM | LC | Independent (Practitioner) | no | Asymmetric  encounter |
| 400.1 | Sondergaard 2015 | | Prioritizing health and psychosocial issues | Negative: quality, actions, impact | MM / PP  SV1  MH | LC/IP | Independent (Practitioner) | no | Asymmetric  encounter |
| 420.1 | Hansen 2015 | | Prioritizing health and psychosocial issues | Negative: quality, actions, impact | MM | LC | Independent (Practitioner or patient) | no | Asymmetric  encounter |
| 420.2 | Hansen 2015 | | Identifying health and psychosocial issues | Negative: quality, actions, impact | MM | LC / IP | Independent (Practitioner) | no | Asymmetric  encounter |
| 634.7 | Gill 2014 | | Identifying health and psychosocial issues | Negative: quality, actions, impact | MM | LC / IP | Independent (Practitioner) | no | Asymmetric  encounter |
| 733.1 | Clarke 2014 | | Discussion topic (in clinical encounter) | Negative: quality, actions, impact | MM  SV2  MH | LC | Independent (Practitioner) | no | Asymmetric  encounter |
| 1098.1 | Luijks 2012 | | Identifying health and psychosocial issues | Negative: quality, actions, impact | MM  MH | No LC or IP issues reported | Independent (Practitioner) | no | Asymmetric  encounter |
| 7088.1 | Barry 2000 | | Discussion topic (Patient conceals info) | Negative: quality, actions, impact | PP  SV1  MH | LC | Independent Patient) | no | Asymmetric  encounter |
| 15346.3 | Cheraghi-Sohi 2013 | | Prioritizing health and psychosocial issues | Negative: actions,  impact  Cannot tell: quality | MM / PP  SV2  MH | No LC or IP issues reported | Independent Patient with practitioner influence) | S6 | Asymmetric  encounter |

Strategy descriptions

- S6: Patient prioritizes conditions that were not controlled by self-management.

## 3.2 Prioritization made in partnership with practitioners during clinical encounters

| **Case ID** | **Studies** | **What** (decision) | **Outcomes**  (quality, actions, impacts) | **Who** (PCCN characteristics) | **Why** (Interactional conditions) | **How** (Independent / partnership) | **Tool or strategy** to facilitate decision | **Decision-making configuration** |
| --- | --- | --- | --- | --- | --- | --- | --- | --- |
| 14.1 | Kangovi 2016 | Health and psychosocial issues management | Positive (quality, actions)  Cannot tell impact | MM  SV1 | No LC or IP issues reported | Partnership  (Patient-Practitioner) | T1 | Well-managed |
| 77.1 | Lyles 2016 | Discussion topic (in clinical encounter) | Positive: quality, actions  Cannot tell impact | MM | No LC or IP issues reported | Partnership  (Patient- Practitioner) | T2 | Well-managed |
| 303.1 | Quinodoz 2016 | Managing health and psychosocial issues | Positive: quality, actions  Cannot tell impact | MM / PP | No LC or IP issues reported | Partnership  (Patient- Practitioner) | S1 | Well-managed |
| 983.1 | Wrede 2013 | Prioritizing health and psychosocial issues | Equivocal: quality  Cannot tell impact or action | MM | No LC or IP issues reported | Partnership  (Patient- Practitioner) | T3 | Well-managed |
| 1045.1 | Stanners 2011 | Identifying health and psychosocial issues | Positive: quality, actions, impact | MM  MH  FU | No LC or IP issues reported | Partnership  (Patient- Practitioner) | S2 | Well-managed |
| 1098.2 | Luijks 2012 | Long term health and psychosocial outcomes | Positive: quality, actions, impact | MM  MH | No LC or IP issues reported | Partnership  (Patient- Practitioner) | S3 | Well-managed |
| 9345.1 | Stokes 2017 | Discussion topic | Positive: quality, actions, impact | MM / PP | No LC or IP issues reported | Partnership  (Patient- Practitioner) | S4 | Well-managed |
| 9356.1 | Summeren 2016 | Long term health and psychosocial outcomes | Positive: quality, actions, impact | MM / PP | No LC or IP issues reported | Partnership  (Patient- Practitioner) | T4 | Well-managed |
| 15885.1 | Loeb 2016 | Managing health and psychosocial issues | Positive: quality, actions, impact | MM  SV1  MH | No LC or IP issues reported | Partnership  (Patient- Practitioner) | S5 | Well-managed |

Tool descriptions

- T1. Prioritization visual aid for patients with low level of health literacy. The aid aimed to prioritize one chronic condition, set a goal for that condition in partnership with primary care practitioners, and then create a patient-driven action plan (self-management)
- T2. Tablet-based prioritization app for patients in the waiting room. The app aimed to help patients select one or two discussion topic priorities for the clinical encounter. Six topics were offered: new issue, old problem, medication, "I need something from the doctor", stress at home or at work, a personal concern or other. After selecting a topic, patients were asked to specify their needs in a textbox.
- T3. “PrefCheck” is a priority checklist for patients and guide for doctors on how to discuss priorities in clinical encounters. The checklist aimed to collect patients’ rating of the perceived importance of their health issues, and to facilitate further patient-doctor communication.
- T4. “OPT conversation tool” aimed to prioritize one health outcome among four during the patient-practitioner encounter (remaining alive, maintaining independence, reducing pain, reducing other symptoms). Patients were told that prioritizing an outcome may affect treatment management and lead to compromise on other outcomes.

Strategy descriptions

- S1. The prioritization should not focus only on medical goals; it should aim for a balance in the management of the diagnosed diseases and the meaning of illness as defined by the patient.
- S2. Promote continuity of care to establish trust between the patient and the practitioner. For the latter, find out about the patient’s context.
- S3. The prioritization should aim for a balance in the management of the diagnosed diseases and the meaning of illness as defined by the patient.
- S4. Negotiate between patient’s priorities and the urgent medical issue. Scheduling multiple encounters to address all priorities and optimise continuity of care. Negotiate compromises with the patient over which aspects of a recommended management plan needed to be adhered.
- S5. Quality of life and functional health of patients should be prioritized.

## 3.3 Prioritization made independently by patients between clinical encounters

| **Case ID** | **Studies** | **What** (decision) | **Outcomes**  (quality, actions, impacts) | **Who** (PCCN characteristics) | **Why** (Interactional conditions) | **How** (Independent / partnership) | **Tool or strategy** (to facilitate decision) | **Decision-making configuration** |
| --- | --- | --- | --- | --- | --- | --- | --- | --- |
| 171.1 | Cheraghi-Sohi 2013 | Prioritizing health and psychosocial issues | Negative: impact  Cannot tell: quality, actions | MM  SV2  MH | LC | Independent (Patient) | S7 | Chaotic |
| 303.2 | Quinodoz 2016 | Tacit treatment prioritization (non-adhere selective) | Equivocal: quality, actions  Cannot tell: impact | MM / PP | No LC or IP issues reported | Independent (Patient) | no | Self-managed |
| 361.1 | Rae 2015 | Health care vs daily life prioritization | Negative: actions,  impact  Cannot tell: quality | SV3  UU | LC | Independent (Patient) | no | Chaotic |
| 562.2 | Coventry 2014 | Prioritizing health vs daily living activities | Negative: quality, actions, impact | MM  SV1  MH | No | Independent (Patient) | no | Self-managed |
| 634.9 | Gill 2014 | Prioritizing health and psychosocial issues | Negative: quality, actions, impact | MM / PP | No | Independent (Patient) | no | Self-managed |
| 3656.1 | O'Donnell 2016 | Prioritizing health vs daily living activities | Negative: actions, impact  Cannot tell: quality | SV3  MH  UU | LC | Independent (Patient) | no | Chaotic |
| 4642.1 | Kuluski 2013 | Prioritizing health and psychosocial issues | Negative: quality  Cannot tell: actions,  impact | MM / PP  SV2  MH | LC | Independent (Patient or practitioner) | no | Chaotic |

Strategy descriptions

- S7: Patient weighs the current and daily impact of some of their long term conditions against those which they perceived could produce more serious and negative outcomes in the future.

## 3.4 Service use decision made independently between clinical encounters

| **Case ID** | **Studies** | | **What** (decision) | **Outcomes**  (quality, actions, impacts) | **Who** (PCCN characteristics) | **Why** (Interactional conditions) | **How** (Independent / partnership) | **Tool or strategy** (to facilitate decision) | **Decision-making configuration** |
| --- | --- | --- | --- | --- | --- | --- | --- | --- | --- |
| 422.1 | Zulman 2015 | | Self-manage health decision and consultation | Negative: impact quality  Cannot tell: actions | MM / PP  FU | LC / IP | Independent (Patient) | S10 | Self-managed |
| 634.3 | Gill 2014 | | Social care services | Negative: quality, actions, impact | MM  MH / SV2 | LC | Independent (Patient) | No | Chaotic |
| 634.4 | Gill 2014 | | Consultation | Negative: quality, actions, impact | MM / PP  MH | LC / IP | Independent (Patient) | No | Chaotic |
| 1445.1 | Claver 2011 | | Emergency use | Negative: quality, actions, impact | MM  SV2  FU | LC | Independent (Patient or caregiver) | No | Chaotic |
| 2110.1 | Themessl-Huber 2007 | | Social care services | Negative: quality, actions, impact | MM  SV2  FU | LC | Independent (Patient) | No | Chaotic |
| 2110.2 | Themessl-Huber 2007 | | Emergency / Hospitalization | Negative: quality, actions, impact | MM  SV2  FU | LC | Independent (Patient) | No | Chaotic |
| 3628.2 | | LaDonna 2016 | Intervention | Negative: quality, actions, impact | MM / PP  SV2 | LC | Independent (Patient) | No | Self-managed |
| 3656.2 | O'Donnell 2016 | | Consultation | Negative: quality, actions, impact | MH /  SV3  UU | LC / IP | Independent (Patient) | No | Chaotic |
| 3833.5 | | Kenning 2013 | Consultation | Negative: impact, actions, quality | MM  SV2/ MH | LC | Independent (Patient) | No | Self-managed |
| 5851.1 | Davis 2009 | | Consultation | Negative: quality, actions, impact | MH /  SV1 | No LC or IP issues reported | Independent (Patient) | S11 | Self-managed |
| 8614.1 | Keene 2004 | | Program engagement | Negative: quality, actions, impact | MM / PP  MH / SV3  UU | IP | Independent (Practitioner) | No | Chaotic |
| 9100.1 | Rabiee 2013 | | Social care services | Negative: quality, actions, impact | MM  SV2 | LC | Independent (Patient) | No | Chaotic |
| 9345.2 | Stokes 2017 | | Program engagement | Negative: quality, actions  Cannot tell: impact | MM / PP | IP | Independent (Patient) | No | Self-managed |
| 9362.1 | Swedberg 2012 | | Home care services | Negative: quality, actions, impact | SV2 | LC / IP | Independent (Patient) | No | Chaotic |
| 12337.1 | Yang 2010 | | Consultation | Negative: quality, actions, impact | MM / PP  SV1 | LC / IP | Independent (Patient) | No | Chaotic |

Strategies description

- S10: Patients identified a number of eHealth technologies they would like to see developed (or adapted) to better support their management of their multiple chronic conditions: (a) uniform medical record platform; (b) resources that consolidate and synthesize online information about multiple chronic conditions, such as apps; (c) high-quality mobile app “bundles” that address their specific conditions, provide assistance with common self-management challenges and integrate information from different health care systems; (d) technology to facilitate communication and coordination with multiple providers across different health care systems; (e) Social support apps and tools.
- S11: Offer specialized support for vulnerable populations to help them to navigate in the health and social services (e.g., social worker as navigator).

## 3.5 Service use decision made independently during clinical encounters

| **Case ID** | **Studies** | **What** (decision) | **Outcomes**  (quality, actions, impacts) | **Who** (PCCN characteristics) | **Why** (Interactional conditions) | **How** (Independent / partnership) | **Tool or strategy** (to facilitate decision) | **Decision-making configuration** |
| --- | --- | --- | --- | --- | --- | --- | --- | --- |
| 146.3 | Risor 2013 | Hospitalisation | Negative: quality, actions, impact | MM  SV2  FU / UU | LC | Independent (Practitioner) | No | Asymmetric  encounter |
| 634.5 | Gill 2014 | Intervention | Negative: quality, actions, impact | MM  SV2 | LC | Independent (Caregiver) | No | Asymmetric  encounter |
| 733.2 | Clarke 2014 | Consultation (reference) | Negative: quality, actions, impact | MM | LC / IP | Independent (Practitioner) | No | Asymmetric  encounter |

## 3.6 Service use decision made in partnership with practitioners during clinical encounters

| **Case ID** | **Studies** | **What** (decision) | **Outcomes**  (quality, actions, impacts) | **Who** (PCCN characteristics) | **Why** (Interactional conditions) | **How** (Independent / partnership) | **Tool or strategy** (to facilitate decision) | **Decision-making configuration** |
| --- | --- | --- | --- | --- | --- | --- | --- | --- |
| 296.1 | Hudon 2015 | Program engagement | Positive: quality, actions, impact | MM  FU | No LC or IP issues reported | Partnership | P1 | Well-managed |
| 1581.1 | Levesque 2010 | Home care | Positive: quality, actions, impact | SV2  UU | No LC or IP issues reported | Partnership | T5 | Well-managed |
| 3628.1 | LaDonna 2016 | Intervention | Positive: quality, actions, impact | MM / PP  SV2 | No LC or IP issues reported | Partnership | S8 | Well-managed |
| 14164.1 | Neal 2000 | Consultation | Positive: quality, actions, impact | MH  FU | No LC or IP issues reported | Partnership | S9 | Well-managed |

Program description

- P1: “V1SAGES Project” is a case management program, implemented by nurses in four family medicine groups in Canada, aiming to establish individualized care plans for patients identified by their family physician as frequent users.

Tool description

- T5: The “Family Caregivers Support Agreement” aimed to facilitate “interpersonal exchanges that promoted a better ﬁt between the views and expectations of practitioners and caregivers” of an aging relative living at home.

Strategy descriptions

- S8. When patients were perceived [by the care team] as being attuned to their symptoms, they could gain the authority to activate care processes as needed rather than following an externally dictated appointment schedule. The decision to refuse treatment could sometimes be legitimized by the care team when they had confidence in the patient's knowledge and ability to make a decision that seems enlightened.
- S9. Individual’s decisions to consult were related to: (1) The perception of the GP role; (2) Past experience of symptoms and consulting; (3) Patient did not want to ‘waste their GP’s time’; (4) Relationship with the GP: (5) Balancing fears; (6) Lay consulting; (7) Comparison with other patients’ consulting experiences; (8) Individual reasons / belief.

## 3.7 Prescription decision made independently by practitioner during clinical encounters

| **Case ID** | **Studies** | **What** (decision) | **Outcomes**  (quality, actions, impacts) | **Who** (PCCN characteristics) | **Why**  (Interactional conditions) | **How** (Independent / partnership) | **Tool or strategy** (to facilitate decision) | **Decision-making configuration** |
| --- | --- | --- | --- | --- | --- | --- | --- | --- |
| 146.1 | Risor 2013 | Prescribe or not | Equivocal: quality, actions, impact | MM / PP  SV2  FU / UU | LC | Independent (Practitioner) | S12 | Asymmetric  encounter |
| 1045.2 | Stanners 2011 | Prescribe or not | Negative: quality, actions, impact | MM / PP  MH /  SV2 | No LC or IP issues reported | Independent (Practitioner) | No | Asymmetric  Encounter |
| 1098.3 | Luijks 2012 | Deprescribe | Negative: quality, actions, impact | MM / PP  MH | IP | Independent (Practitioner) | No | Asymmetric  encounter |
| 3590.1 | Mc Namara 2016 | Deprescribe | Negative: quality, actions, impact | MM / PP | IP | Independent (Practitioner) | No | Asymmetric  encounter |
| 3590.2 | Mc Namara 2016 | Prescribe or not | Negative: quality, actions, impact | MM / PP | IP | Independent (Practitioner) | No | Asymmetric  encounter |
| 3594.1 | Puts 2016 | Clinical decision–making | Negative: quality, impact  Cannot tell: actions | MM  SV2 | LC / IP | Independent (Specialist) | No | Asymmetric  encounter |
| 3833.1 | Kenning 2013 | Prescribe or not | Negative: quality, actions, impact | MM / PP  MH / SV2 | No LC or IP issues reported | Independent (Practitioner) | No | Asymmetric  encounter |
| 16192.1 | Robben 2012 | Deprescribe and clinical decision making | Negative: quality, actions, impact | MM / PP  MH /  SV2 | LC / IP | Independent (Practitioner) | S13 | Asymmetric  encounter |

Strategy descriptions

- S12: Ideally, the practitioner’s decision to prescribe antibiotics or steroids for COPD patients has to follow a medically informed and patient-centred decision-making including the following considerations: (a) whether prescribing upper limits of medication dosages was acceptable and for how long; (b) when it was rational to prescribe antibiotics or steroids according to clinical ﬁndings and history; (c) how patients would accept the medication they prescribed; (d) whether they would be compliant, specifically regarding the up-take and purchase of the medication; and (e) whether the patient would be able to have a dialogue about effects and use with their physician.
- S13: Participants used several strategies to enhance the quality or amount of information provided by professionals such bringing someone to the doctor’s appointment to receive more information, or preparing the clinical encounter (e.g., by making a list of questions in advance and making sure these were discussed during their visit), or searching for information on their own (either in advance to prepare for the encounter, or afterwards to seek additional information about the topics discussed or services available) using all kinds of sources including the internet and patient organizations, or reporting things mentioned by another specialist.

## 3.8 Prescription decision made independently by patient between clinical encounters

| **Case ID** | **Studies** | | **What** (decision) | **Outcomes**  (quality, actions, impacts) | **Who** (PCCN characteristics) | **Why** (Interactional conditions) | **How** (Independent / partnership) | **Tool or strategy** (to facilitate decision) | **Decision-making configuration** |
| --- | --- | --- | --- | --- | --- | --- | --- | --- | --- |
| 1310.1 | Mishra 2011 | Adhere or not | | Negative: (quality, actions, impact | MM / PP  MH / SV1 | LC / IP | Independent (Patient) | No | Chaotic |
| 2149.1 | Mukherjee 2006 | Adhere or not | | Negative: quality, actions, impact | MM / PP  MH / SV1 | IP | Independent (Patient) | No | Chaotic |
| 3709.1 | Wells 2011(Wells et al., 2011) | Adhere or not | | Negative: quality, actions, impact | MM / PP  MH / SV1 | LC | Independent (Patient) | No | Chaotic |
| 3738.1 | Glasser 2016 | Adhere or not | | Negative: quality, actions, impact | MM / PP  MH / SV2 | LC / IP | Independent (Patient) | No | Chaotic |

## 3.9 Behavior Change decision made independently by patient between clinical encounters

| **Case ID** | **Studies** | **What** (decision) | **Outcomes**  (quality, actions, impacts) | **Who** (PCCN characteristics) | **Why**  (Interactional conditions) | **How** (Independent / partnership) | **Tool or strategy** (to facilitate decision) | **Decision-making configuration** |
| --- | --- | --- | --- | --- | --- | --- | --- | --- |
| 146.2 | Risor 2013 | Smoking, Alcohol, No physical activity | Negative: quality, actions, impact | SV1 & SV2  FU / UU | LC | Independent (Patient) | No | Chaotic |
| 583.1 | Aschbrenner 2014 | Non-healthy eating & sedentarity | Negative: quality, actions, impact | MH  SV2 | LC | Independent (Caregiver) | No | Chaotic |
| 634.10 | Gill 2014 | Continue To Drive | Negative: quality, actions, impact | MH  SV2 | LC / IP | Independent (Patient) | No | Chaotic |
| 3738.2 | Glasser 2016 | Smoking | Negative: quality, actions, impact | MM / PP  MH / SV2 | LC / IP | Independent (Patient) | No | Chaotic |

## 3.10 Institutionalization decision made independently by caregiver between clinical encounters

| **Case ID** | **Studies** | **What** (decision) | **Outcomes**  (quality, actions, impacts) | **Who** (PCCN characteristics) | **Why**  (Interactional conditions) | **How** (Independent / partnership) | **Tool or strategy** (to facilitate decision) | **Decision-making configuration** |
| --- | --- | --- | --- | --- | --- | --- | --- | --- |
| 1784.1 | Schoenmakers 2009 | Move to a care facility | Negative: quality, actions, impact | MM  MH / SV2 | FC | Independent (Caregiver) | No | Chaotic |
| 1982.1 | Belleau 2007 | Move to a care facility | Negative: quality, actions, impact | MH / SV2 | FC | Independent (Caregiver or patient) | S14 | Chaotic |
| 2965.1 | Hicks 1999 | Move to a care facility | Negative: quality, actions, impact | MH / SV2 | FC  CC | Independent (Caregiver) | S15 | Chaotic |
| 4642.2 | Kuluski 2013 | Move to a care facility | Negative: quality, actions, impact | MM  MH / SV2 | FC | Patient | No | Chaotic |
| 8153.1 | Chene 2006 | Move to a care facility | Negative: quality, actions, impact | MH / SV2 | FC  / CC | Independent (Caregiver) | No | Chaotic |

Strategy descriptions

- S14: Four distinct strategies were adopted by professionals and family members when an older person refused to be rehoused and no other solution could be found: (a) Transparency was the most ethically desirable, although it had significant limitations in practice ; (b) Often subterfuge was used to institutionalize people against their will (this occurred through a traumatic process, often provoking remorse among caregivers); (c) Mitigation strategies, which consisted of trying first to convince the elderly person and then, in the event of failure, using subterfuge; (d) The limit waiting, which consisted of waiting for an emergency situation to institutionalize the person.
- S15: Alliances and divisions were created between stakeholders to influence the decision to institutionalize or maintain home care. In addition to considering health issues, hygiene and behavioral issues led stakeholders to privilege institutionalization. Others, advocating community and family values (cultural influence), preferred that family takes care of the relative with dementia.
