# Supplementary material for: Decision-making and related outcomes of patients with complex care needs in primary care settings: a systematic literature review with a case-based qualitative synthesis
Source: BMC Prim Care. 2022 Nov 9;23:279. doi: 10.1186/s12875-022-01879-5 (PMC9644584; doi:10.1186/s12875-022-01879-5)
Supplement: Supplementary file 4 — Additional file 4. Quality appraisal of the 47 included studies. [file 12875_2022_1879_MOESM4_ESM.docx]

# Additional file 4 - Quality appraisal of the 47 included studies

| **Studies** | **Criteria from the Mixed Methods Appraisal Tool** | | | | | | | | | | | | | | | | | | | |
| --- | --- | --- | --- | --- | --- | --- | --- | --- | --- | --- | --- | --- | --- | --- | --- | --- | --- | --- | --- | --- |
|  | **1.1** | **1.2** | **1.3** | **1.4** | **1.5** | **3.1** | **3.2** | **3.3** | **3.4** | **3.5** | **4.1** | **4.2** | **4.3** | **4.4** | **4.5** | **5.1** | **5.2** | **5.3** | **5.4** | **5.5** |
| Aschbrenner et al. (2014) | 0 | 1 | 1 | 1 | 1 |  |  |  |  |  | 1 | 0 | 1 | 1 | 1 | 1 | 1 | 1 | 1 | 1 |
| Barry et al. (2000) | 0 | 1 | 1 | 1 | 1 |  |  |  |  |  |  |  |  |  |  |  |  |  |  |  |
| Belleau (2007) | 0 | 1 | 1 | 1 | 1 |  |  |  |  |  |  |  |  |  |  |  |  |  |  |  |
| Chene (2006) | 0 | 1 | 1 | 1 | 1 |  |  |  |  |  | 1 | 1 | 1 | 1 | 1 | 1 | 1 | 1 | 1 | 1 |
| Cheraghi-Sohi et al. (2013a) | 1 | 1 | 1 | 1 | 1 |  |  |  |  |  |  |  |  |  |  |  |  |  |  |  |
| Cheraghi-Sohi et al. (2013b) | 1 | 1 | 1 | 1 | 1 |  |  |  |  |  |  |  |  |  |  |  |  |  |  |  |
| Clarke et al. (2014) | 0 | 1 | 1 | 1 | 1 |  |  |  |  |  |  |  |  |  |  |  |  |  |  |  |
| Claver (2011) | 0 | 1 | 1 | 1 | 1 |  |  |  |  |  |  |  |  |  |  |  |  |  |  |  |
| Coventry et al. (2014) | 0 | 1 | 1 | 1 | 1 |  |  |  |  |  |  |  |  |  |  |  |  |  |  |  |
| Davis et al. (2009) | 0 | 1 | 0 | 1 | 0 |  |  |  |  |  |  |  |  |  |  |  |  |  |  |  |
| Gill et al. (2014) | 1 | 1 | 1 | 1 | 1 |  |  |  |  |  |  |  |  |  |  |  |  |  |  |  |
| Glasser et al. (2016) | 0 | 1 | 1 | 1 | 1 |  |  |  |  |  |  |  |  |  |  |  |  |  |  |  |
| Hansen et al. (2015) | 0 | 1 | 1 | 1 | 1 |  |  |  |  |  |  |  |  |  |  |  |  |  |  |  |
| Hicks and Lam (1999) | 1 | 1 | 0 | 1 | 0 |  |  |  |  |  |  |  |  |  |  |  |  |  |  |  |
| Hudon et al. (2015) | 1 | 1 | 1 | 1 | 1 |  |  |  |  |  |  |  |  |  |  |  |  |  |  |  |
| Kangovi et al. (2016) |  |  |  |  |  |  |  |  |  |  | 0 | 0 | 1 | 1 | 1 |  |  |  |  |  |
| Keene et al. (2004) | 0 | 1 | 1 | 1 | 1 |  |  |  |  |  |  |  |  |  |  |  |  |  |  |  |
| Kenning et al. (2013) | 0 | 1 | 1 | 1 | 1 |  |  |  |  |  |  |  |  |  |  |  |  |  |  |  |
| Kuluski et al. (2013) | 1 | 1 | 1 | 1 | 1 |  |  |  |  |  |  |  |  |  |  |  |  |  |  |  |
| LaDonna et al. (2016) | 1 | 1 | 1 | 1 | 1 |  |  |  |  |  |  |  |  |  |  |  |  |  |  |  |
| Lévesque et al. (2010) | 0 | 1 | 1 | 1 | 1 |  |  |  |  |  |  |  |  |  |  |  |  |  |  |  |
| Loeb et al. (2016) | 1 | 1 | 1 | 1 | 1 |  |  |  |  |  |  |  |  |  |  |  |  |  |  |  |
| Luijks et al. (2012) | 0 | 1 | 1 | 1 | 1 |  |  |  |  |  |  |  |  |  |  |  |  |  |  |  |
| Lyles et al. (2016) | 1 | 1 | 1 | 1 | 1 |  |  |  |  |  |  |  |  |  |  |  |  |  |  |  |
| Mc Namara et al. (2016) | 0 | 1 | 1 | 1 | 1 |  |  |  |  |  |  |  |  |  |  |  |  |  |  |  |
| Mishra et al. (2011) | 0 | 1 | 1 | 1 | 1 |  |  |  |  |  |  |  |  |  |  |  |  |  |  |  |
| Mukherjee et al. (2006) | 0 | 1 | 1 | 1 | 1 |  |  |  |  |  |  |  |  |  |  |  |  |  |  |  |
| Neal et al. (2000) | 0 | 1 | 1 | 1 | 1 |  |  |  |  |  |  |  |  |  |  |  |  |  |  |  |
| O'Donnell et al. (2016) | 1 | 1 | 1 | 1 | 1 |  |  |  |  |  |  |  |  |  |  |  |  |  |  |  |
| Paskins et al. (2015) | 0 | 1 | 1 | 0 | 1 |  |  |  |  |  |  |  |  |  |  |  |  |  |  |  |
| Puts et al. (2016) | 0 | 1 | 1 | 1 | 1 |  |  |  |  |  | 1 | 1 | 1 | 0 | 1 | 0 | 1 | 1 | 1 | 1 |
| Quinodoz et al. (2016) | 0 | 1 | 1 | 1 | 1 |  |  |  |  |  |  |  |  |  |  |  |  |  |  |  |
| Rabiee (2013) | 0 | 1 | 1 | 1 | 1 |  |  |  |  |  |  |  |  |  |  |  |  |  |  |  |
| Rae and Rees (2015) | 1 | 1 | 1 | 1 | 1 |  |  |  |  |  |  |  |  |  |  |  |  |  |  |  |
| Risor et al. (2013) | 1 | 1 | 1 | 1 | 1 |  |  |  |  |  |  |  |  |  |  |  |  |  |  |  |
| Robben et al. (2012) | 1 | 1 | 1 | 1 | 1 |  |  |  |  |  |  |  |  |  |  |  |  |  |  |  |
| Schoenmakers et al. (2009) |  |  |  |  |  | 1 | 0 | 1 | 1 | 1 |  |  |  |  |  |  |  |  |  |  |
| Sondergaard et al. (2015) | 0 | 1 | 1 | 1 | 1 |  |  |  |  |  |  |  |  |  |  |  |  |  |  |  |
| Stanners et al. (2012) | 1 | 1 | 1 | 1 | 1 |  |  |  |  |  |  |  |  |  |  |  |  |  |  |  |
| Stokes et al. (2017) | 0 | 1 | 1 | 1 | 1 |  |  |  |  |  |  |  |  |  |  |  |  |  |  |  |
| Summeren et al. (2016) | 0 | 1 | 1 | 1 | 1 |  |  |  |  |  | 1 | 1 | 0 | 1 | 1 | 1 | 0 | 1 | 0 | 1 |
| Swedberg et al. (2012) | 1 | 1 | 1 | 1 | 1 |  |  |  |  |  |  |  |  |  |  |  |  |  |  |  |
| Themessl-Huber et al. (2007) | 0 | 1 | 1 | 1 | 1 |  |  |  |  |  |  |  |  |  |  |  |  |  |  |  |
| Wells et al. (2011) | 0 | 1 | 1 | 1 | 1 |  |  |  |  |  |  |  |  |  |  |  |  |  |  |  |
| Wrede et al. (2013) | 0 | 1 | 1 | 1 | 1 |  |  |  |  |  |  |  |  |  |  |  |  |  |  |  |
| Yang et al. (2010) | 0 | 1 | 1 | 1 | 1 |  |  |  |  |  |  |  |  |  |  |  |  |  |  |  |
| Zulman et al. (2015) | 0 | 1 | 1 | 1 | 1 |  |  |  |  |  |  |  |  |  |  |  |  |  |  |  |

0 = criterion not met or cannot tell; 1 = criterion met

MMAT Criteria ([mixedmethodsappraisaltoolpublic/MMAT-2018-criteria-and-manual.pdf](http://mixedmethodsappraisaltoolpublic.pbworks.com/w/file/127916259/MMAT_2018_criteria-manual_2018-08-01_ENG.pdf))

*1. Qualitative research or qualitative component of a mixed methods study:*

1.1. Is the qualitative approach appropriate to answer the research question?; 1.2. Are the qualitative data collection methods adequate to address the research question?; 1.3. Are the findings adequately derived from the data?; 1.4. Is the interpretation of results sufficiently substantiated by data?; 1.5. Is there coherence between qualitative data sources, collection, analysis and interpretation?

*3. Quantitative non-randomized study (NRS) or NRS component of a mixed methods study:*

3.1. Are the participants representative of the target population?; 3.2. Are measurements appropriate regarding both the outcome and intervention (or exposure)?; 3.3. Are there complete outcome data?; 3.4. Are the confounders accounted for in the design and analysis?; 3.5. During the study period, is the intervention administered (or exposure occurred) as intended?

4. *Quantitative descriptive study or corresponding component of a mixed methods study:*

4.1. Is the sampling strategy relevant to address the research question?; 4.2. Is the sample representative of the target population?; 4.3. Are the measurements appropriate?; 4.4. Is the risk of nonresponse bias low?; 4.5. Is the statistical analysis appropriate to answer the research question?

*5. Mixed methods study:*

5.1. Is there an adequate rationale for using a mixed methods design to address the research question?; 5.2. Are the different components of the study effectively integrated to answer the research question?; 5.3. Are the outputs of the integration of qualitative and quantitative components adequately interpreted?; 5.4. Are divergences and inconsistencies between quantitative and qualitative results adequately addressed? ; 5.5. Do the different components of the study adhere to the quality criteria of each tradition of the methods involved?
