# Supplementary material for: Decision-making and related outcomes of patients with complex care needs in primary care settings: a systematic literature review with a case-based qualitative synthesis
Source: BMC Prim Care. 2022 Nov 9;23:279. doi: 10.1186/s12875-022-01879-5 (PMC9644584; doi:10.1186/s12875-022-01879-5)
Supplement: Supplementary file 5 — Additional file 5. Treatment decision-making models. [file 12875_2022_1879_MOESM5_ESM.docx]

**Additional file 5 Treatment decision-making models**

In their seminal papers Charles and Gafni (1, 2) conceptualized shared decision-making model (SDM) and described other treatment decision-making models. In the paternalistic model, the patient plays a passive role in the treatment decision-making process vis-a-vis the physician, who is seen as the expert and “guardian of the patient's best interest” (1: p.682-83). The role of the patient is limited to providing consent to the treatment (3). Three main models have since been proposed in reaction to the paternalistic model.

**First - Professional-as-agent model**

In this model, deriving from the agency model in health economics, the physician directs health care as the patient’s agent, making choices for them on the assumption that they understand what their patients want (4). One of the central critiques of this model is that physicians act on this assumption without first explicitly testing it (1: p.684)*.* Medical anthropology studies also suggest that the PCCN’s perception of his/her illness episode changes according to context, emotional state, cultural values and taboos, and intersubjectivity in clinical encounters (5, 6). During the clinical encounter, however, the practitioner only has access to one version of the patient's illness perception.

**Second - Informed decision-making model**

This model puts the responsibility of the decision on the patient (4). The patient becomes autonomous once empowered by being informed of the possible risks of alternative therapeutic options and their clinical effectiveness, and can make decisions that reflect both their preferences and the best scientific knowledge available (7, cited in 1: p.683). In this model, the physician’s role is limited to giving information to the patient, who theoretically no longer needs to share the decision-making because the patient now possesses both components (information and preferences) viewed as essential to the task (8). In this case, “information transfer can be done without the presence of any health care worker, for example, by the patient viewing an interactive video” (1: p.683). In this model, decision aids are a form of educational intervention, presenting information to patients about treatment benefits and risks to “encourage evidence-based decision-making” (2: p.655). Gafni et al. (4: p.352) argue “that the approach of transferring information to the patient is easier (but not easy) and, hence, more feasible than transferring each patient's preferences to the physician in each medical encounter.” These models both involve information sharing, but neither explicitly involves shared decision-making.

**Third – Shared decision-making model**

SDM model advocates for a process where patient and practitioner work together to make an informed choice congruent with patient values (9, 10). This model “is seen as a mechanism to decrease the informational and power asymmetry between doctors and patients by increasing patients' information, sense of autonomy and/or control over treatment decisions that affect their well-being” (1: p 682). Charles et al. (1) suggest that in SDM, (a) at least two participants, i.e., a clinician and a patient, are involved; (b) both parties share information; (c) both parties take steps to build a consensus about the preferred treatment; and (d) an agreement is reached on the treatment to implement. They subsequently revised this model to incorporate a more dynamic perspective on shared decision-making by recognizing the iterative nature of this process (2).

SDM has been associated with improving well-being and better experiences of the health and social care system (9-13). The SDM model has been revisited and adapted multiple times, e.g. to chronic disease (12) and primary care (13). Nonetheless, PCCNs’ decisional needs have not been considered in any of these adaptations. In this paper we revisit Charles et al.’s model and pay closer attention to what it implies for the specific needs of PCCNs.

SDM is not suitable for every patient, decision, and context. Some patients face uncertainty about their disease outcome and feel unwelcome time pressures to make a choice among competing alternatives. Extreme psychological and/or physiological vulnerability may make it difficult for patients to participate in decision-making, no matter how well informed they feel (1: p. 684). Research indicates that SDM and thus effective support for decision-making is not perceived by the most vulnerable populations (14). However, practising SDM with vulnerable populations can reduce inequities in health (15). Although Charles et al. recognize that different models of decision-making are more or less appropriate depending on the context, they do not discuss this in depth. Faced with the enormous task of contextualizing decision-making, the authors focus on one-on-one clinical encounters, to the detriment of more complex context such as decision making with interprofessional teams (16). While interprofessional SDM has been addressed since (17), the contextualization of this model with the PCCNs population has received little attention.

1. Charles C, Gafni A, Whelan T. Shared decision-making in the medical encounter: what does it mean? (or it takes at least two to tango). Soc Sci Med. 1997;44(5):681-92.

2. Charles C, Gafni A, Whelan T. Decision-making in the physician-patient encounter: revisiting the shared treatment decision-making model. Soc Sci Med. 1999;49(5):651-61.

3. Emanuel EJ, Emanuel LL. Four models of the physician-patient relationship. JAMA. 1992;267(16):2221-6.

4. Gafni A, Charles C, Whelan T. The physician-patient encounter: the physician as a perfect agent for the patient versus the informed treatment decision-making model. Soc Sci Med. 1998;47(3):347-54.

5. Bujold M. Le patient intégrateur: analyse de l’articulation d’une pluralité de voix / voies dans une clinique intégrative québécoise. Ste-Foy: Université Laval; 2011.

6. Bujold M. Ethnomedical ethics with regard to patient plurivocality: between autonomy and heteronomy. Journal International de Bioéthique. 2015;26(4):19-36.

7. Hurley J, Birch S, Eyles J. Information, efficiency and decentralization within health care systems. CHEPA Working Paper 92-21; McMaster University, Hamilton.1992.

8. Levine MN, Gafni A, Markham B, MacFarlane D. A bedside decision instrument to elicit a patient's preference concerning adjuvant chemotherapy for breast cancer. Ann Intern Med. 1992;117(1):53-8.

9. Legare F, Stacey D, Turcotte S, Cossi MJ, Kryworuchko J, Graham ID, et al. Interventions for improving the adoption of shared decision making by healthcare professionals. Cochrane Database Syst Rev. 2014(9):CD006732.

10. Melbourne E, Roberts S, Durand MA, Newcombe R, Legare F, Elwyn G. Dyadic OPTION: Measuring perceptions of shared decision-making in practice. Patient Educ Couns. 2011;83(1):55-7.

11. Légaré F, Adekpedjou R, Stacey D, Turcotte S, Kryworuchko J, Graham ID, et al. Interventions for increasing the use of shared decision making by healthcare professionals. Cochrane Database Syst Rev. 2018(7).

12. Wieringa TH, Rodriguez-Gutierrez R, Spencer-Bonilla G, de Wit M, Ponce OJ, Sanchez-Herrera MF, et al. Decision aids that facilitate elements of shared decision making in chronic illnesses: a systematic review. Syst Rev. 2019;8(1):121.

13. Murray E, Charles C, Gafni A. Shared decision-making in primary care: tailoring the Charles et al. model to fit the context of general practice. Patient Educ Couns. 2006;62(2):205-11.

14. Haesebaert J, Adekpedjou R, Croteau J, Robitaille H, Legare F. Shared decision-making experienced by Canadians facing health care decisions: a Web-based survey. CMAJ Open. 2019;7(2):E210-E6.

15. Durand MA, Carpenter L, Dolan H, Bravo P, Mann M, Bunn F, et al. Do interventions designed to support shared decision-making reduce health inequalities? A systematic review and meta-analysis. PLoS One. 2014;9(4):e94670.

16. Gaboury I, Bujold M, Boon H, Moher D. Interprofessional collaboration within Canadian integrative healthcare clinics: Key components. Soc Sci Med. 2009;69(5):707-15.

17. Legare F, Stacey D, Pouliot S, Gauvin FP, Desroches S, Kryworuchko J, et al. Interprofessionalism and shared decision-making in primary care: a stepwise approach towards a new model. Journal of interprofessional care. 2011;25(1):18-25.
